# A self-perpetuating neuron-intrinsic GSDMD–mtDNA–AIM2 inflammasome axis drives neuronal pyroptosis and cognitive impairment after traumatic brain injury

**DOI:** 10.3389/fimmu.2026.1867920

**Published:** 2026-06-19

**Authors:** Tian Li, Siyu Huang, Junjun Zhang, Xueer Liu, Lihong Zhu, Yue Li, Runmin Lin, Xiaoxuan Chen, Kangsheng Li, Weiqiang Chen, Jiangtao Sheng

**Affiliations:** 1 Department of Neurosurgery, The First Affiliated Hospital of Shantou University Medical College, Shantou, Guangdong, China; 2 Department of Microbiology and Immunology, Shantou University Medical College, Shantou, Guangdong, China; 3 The Department of International Medical Services, the Affiliated Cancer Hospital of Shantou University Medical College, Guangdong, China

**Keywords:** AIM2 inflammasome, cognitive impairment, GSDMD, mtDNA, traumatic brain injury

## Abstract

**Introduction:**

Traumatic brain injury (TBI) is a leading cause of mortality and long-term disability worldwide, producing acute neurological deficits and lasting cognitive impairment. Post-injury neuroinflammation is a principal driver of secondary damage and contributes substantially to TBI-induced cognitive dysfunction (TBI-CD), yet the cell-autonomous mechanisms operating within neurons remain incompletely characterised. The AIM2 inflammasome — a cytosolic sensor of double-stranded DNA that drives pro-inflammatory cytokine release and pyroptosis — has been studied primarily in myeloid cells, and its role within neurons after TBI is unclear.

**Methods:**

Here, using a controlled cortical impact (CCI) mouse model, an *in vitro* mechanical-injury model, AAV-mediated neuron-specific AIM2 knockdown and a comprehensive set of behavioural and molecular assays, we define a neuron-intrinsic, self-perpetuating GSDMD–mtDNA–AIM2 axis that drives neuronal pyroptosis and cognitive decline after TBI.

**Results:**

CCI triggered acute (24 h) AIM2 inflammasome activation specifically in cortical and hippocampal neurons, neuronal pyroptosis and CA3 neuronal loss. AAV-mediated knockdown of AIM2 in hippocampal CA3 neurons significantly reduced neuronal loss in this region and rescued cognitive performance in the Y-maze, novel object recognition and Morris water maze at 14 and 28 days post-injury. Mechanistically, mechanical injury caused early (3–6 h) release of mitochondrial DNA (mtDNA) — but not nuclear DNA — into the neuronal cytosol, where it directly activated the AIM2 inflammasome and engaged caspase-1/GSDMD-dependent pyroptosis; ethidium bromide-mediated mtDNA depletion reversed each pyroptotic marker. Within the first 0.5–3 h post-injury, activated GSDMD N-terminal fragments (GSDMD-NT) translocated to mitochondria, disrupted mitochondrial membrane potential (ΔΨm) and promoted further mtDNA leakage; CRISPR knockout of GSDMD, but not of Bax, prevented this injury-induced mitochondrial dysfunction. Delayed pharmacological inhibition of caspase-1 (VX-765 applied 6 h after injury, after the first wave) selectively suppressed a discrete second wave of cytosolic mtDNA release at 9–12 h and attenuated late-phase LDH release, providing direct experimental evidence for a self-perpetuating second wave of mtDNA-driven AIM2 activation.

**Conclusion:**

These findings define a self-perpetuating, neuron-intrinsic GSDMD–mtDNA–AIM2 inflammasome–pyroptosis axis as a driver of TBI-CD, and identify neuron-targeted AIM2 silencing as a candidate therapeutic strategy for limiting post-TBI neuroinflammation and cognitive decline.

## Introduction

Traumatic brain injury (TBI) is a major global health burden and a frequent cause of long-term neurological sequelae, including persistent cognitive dysfunction (1). Neuroinflammation has emerged as a central mediator of the secondary injury cascades that follow the initial impact, driving neuronal loss and contributing substantially to chronic cognitive impairment (2). Defining the specific molecular drivers and cell types involved in this response is therefore essential for developing effective therapeutic strategies.

Inflammasomes — multiprotein complexes that sense danger signals and initiate inflammatory responses — are key orchestrators of post-TBI neuroinflammation. Much attention has focused on the NLRP3 inflammasome, which is activated mainly in microglia and astrocytes and promotes the maturation and release of pro-inflammatory cytokines such as IL-1β and IL-18, contributing substantially to the inflammatory milieu (3, 4). The inflammasome landscape in TBI is, however, broader than NLRP3 alone: the neuronal NLRP1 inflammasome is rapidly activated by mechanical stress-induced K^+^ efflux and pannexin-1 channel opening (or protease cleavage), leading to caspase-1-dependent pyroptosis and early neuronal damage (5, 6). The AIM2 inflammasome — a cytosolic sensor of double-stranded DNA (dsDNA) that is notably enriched in neurons (7, 8) — has received comparatively little attention in TBI. Canonically, AIM2 binds cytosolic dsDNA and recruits the adaptor protein ASC, activating caspase-1, driving Gasdermin D (GSDMD) pore formation, and processing IL-1β and IL-18 (9). Although crucial for host defence, dysregulated AIM2-mediated pyroptosis in the neuronal compartment after TBI could represent an important, yet underappreciated, driver of neuronal death and sustained neuroinflammation (10, 11). The specific contribution of neuron-intrinsic AIM2 signaling to TBI pathology, distinct from glial inflammasome pathways, remains poorly defined.

Beyond AIM2 and NLRP3, several non-inflammasome DNA-sensing pathways could exacerbate secondary damage but appear to act downstream of, or in parallel to, AIM2 in neurons. After mitochondrial membrane permeabilization, aberrant accumulation of mitochondrial DNA (mtDNA) in the cytosol acts as a potent damage-associated molecular pattern (DAMP). The sensing of such ectopic DNA involves several pattern recognition receptors (PRRs). For example, the cyclic GMP-AMP synthase (cGAS)–stimulator of interferon genes (STING) pathway is a well-known sensor of cytosolic DNA and primarily drives type I interferon (IFN-I) responses and sterile inflammation (12). Toll-like receptor 9 (TLR9) can recognize CpG-rich mtDNA, but typically requires endosomal uptake of extracellular DNA rather than direct sensing of intracellular leakage (13). Although these pathways contribute to the broader inflammatory milieu, the AIM2 inflammasome occupies a distinct niche: it directly surveys the cytosol and, upon binding dsDNA, nucleates ASC to drive rapid caspase-1 activation and GSDMD-mediated pyroptosis (14). cGAS–STING and TLR9 are more commonly engaged in immune cells such as microglia and macrophages (15, 16), whereas in the TBI context cytosolic dsDNA in neurons appears more likely to activate the AIM2 inflammasome (17, 18). Unlike the transcriptional IFN response driven by cGAS, AIM2 signaling produces immediate lytic cell death. In the setting of TBI-induced neuronal loss, AIM2 thus appears to act as the predominant executioner, distinguishing it from the other DNA-sensing mechanisms.

A critical gap remains in understanding the upstream triggers and mechanisms governing neuronal AIM2 activation in response to the primary mechanical forces inherent to TBI. How mechanical stress translates into the required cytosolic dsDNA ligand within neurons is unclear. Given their sensitivity to injury and abundant dsDNA content, mitochondria are plausible sources, and mtDNA leakage could serve as the initiating danger signal for neuronal AIM2. The mechanism of mtDNA release itself also warrants investigation. Beyond its role as the terminal executioner of pyroptosis at the plasma membrane, the GSDMD N-terminal fragment (GSDMD-NT) can also directly target and permeabilize mitochondrial membranes (14, 19, 20). This raises the possibility of a self-perpetuating amplification loop in early TBI: initial mechanical stress drives some GSDMD activation, which damages mitochondria and releases mtDNA, further activating the AIM2–caspase-1–GSDMD axis and amplifying neuronal pyroptosis and the local inflammatory response. Defining the GSDMD–mitochondria interplay in the context of TBI-induced neuronal AIM2 activation is therefore important. Addressing these mechanistic questions is timely because current anti-inflammatory strategies typically lack cell-type specificity, highlighting the need for targeted approaches such as AAV-mediated silencing in hippocampal neurons to dissect and potentially modulate this pathway (21, 22).

We therefore hypothesized that TBI-induced mechanical injury initiates a neuron-intrinsic inflammatory cascade comprising early GSDMD-NT-mediated mitochondrial permeabilization, subsequent mtDNA release into the cytosol, activation of the AIM2 inflammasome, and execution of pyroptosis, ultimately contributing to hippocampal neuronal loss and associated cognitive deficits. To test this hypothesis we (i) characterized AIM2 inflammasome activation via mtDNA leakage in neurons after mechanical injury *in vivo* and *in vitro*, (ii) investigated an early, potentially direct, role for GSDMD in mitochondrial dysfunction and mtDNA release, and (iii) determined the therapeutic potential of targeted AIM2 knockdown in hippocampal neurons for rescuing TBI-induced cognitive impairment. The findings define a neuron-enriched pyroptotic pathway that contributes to TBI neuroinflammation and cognitive dysfunction, and identify a potential target for future neuroprotective therapies.

## Materials and methods

### Animals and controlled cortical impact model

All experiments used male C57BL/6 mice (8–10 weeks old, 20–25 g) obtained from the Experimental Animal Center of Shantou University. Mice were housed under standard conditions (12-h light/dark cycle, 22–24°C, ad libitum food and water) and randomized to groups. The moderate CCI model was performed as previously described (23). Mice were anaesthetized with isoflurane (3–4% for induction, 1.5–2% for maintenance). A 4-mm-diameter craniotomy was performed over the left parietal cortex (centered at AP −2.0 mm, ML −2.0 mm relative to bregma). A pneumatic impactor with a 3-mm flat tip delivered a single impact (velocity, 3 m/s; depth, 1.5 mm below the dura; dwell time, 500 ms). The bone flap was not replaced, and the scalp was sutured. Postoperatively, mice received subcutaneous buprenorphine (0.1 mg/kg) for analgesia and were monitored daily for weight loss and general health. Sham-operated controls underwent identical surgical procedures, including craniotomy, but without impact. Group sizes were n = 8–12 mice per condition for behavioral and histological assays and n = 3–5 for molecular analyses (Western blot and qPCR); exact n values are specified in the figure legends. Data are presented as mean ± SEM or mean ± SD, with 95% confidence intervals where applicable.

### Adeno-associated virus production and stereotaxic injection

Recombinant AAV vectors (serotype AAV-PhP.eB, selected for efficient CNS neuronal transduction) expressing either AIM2-shRNA (target sequence 5′-GAAAGAAGCTGAACGTAAACT-3′) or a non-targeting scramble shRNA (5′-GATGTTTCGAGAGGAGGACTA-3′) under the human U6 promoter were generated. Plasmids were packaged by triple transfection of AAV-293 cells, and viral particles were purified by iodixanol gradient ultracentrifugation, with titers of ≥ 1 × 10¹³ vector genomes (vg) per mL.

Two weeks before CCI, mice were anaesthetized as above and placed in a stereotaxic frame (Shanghai XinRuan). A total of 2 μL of AAV solution (titer ≈ 1 × 10¹³ vg/mL) was injected unilaterally into the left hippocampal CA3 region using the coordinates AP −2.0 mm, ML + 1.8 mm (relative to bregma), DV −1.9 mm (from the dura). Injections were performed at 0.1 μL/min with a Hamilton syringe fitted with a 33-gauge needle. The needle was left in place for 5 min after injection to limit reflux and was then slowly withdrawn. Mice recovered for 14 days before CCI surgery to allow optimal viral expression.

### Neurological severity score

Neurological deficits were assessed using a 10-point NSS scale. Tests included motor tasks (seeking behavior, beam balance and grip strength), sensory tests and reflex tests. Scoring was performed by an investigator blinded to the experimental groups at baseline (pre-injury) and at 1, 3, 7, 14 and 28 days post-CCI. Higher scores indicate greater neurological impairment (maximum score = 10).

### Behavioral testing

All behavioral tests were performed during the light cycle by an investigator blinded to treatment groups. Before the cognitive tests, mice were confirmed to be free of motor and visual deficits by NSS and swim-speed assessment. Mice were habituated to the testing room for at least 30 min before each test.

**Figure 1:**
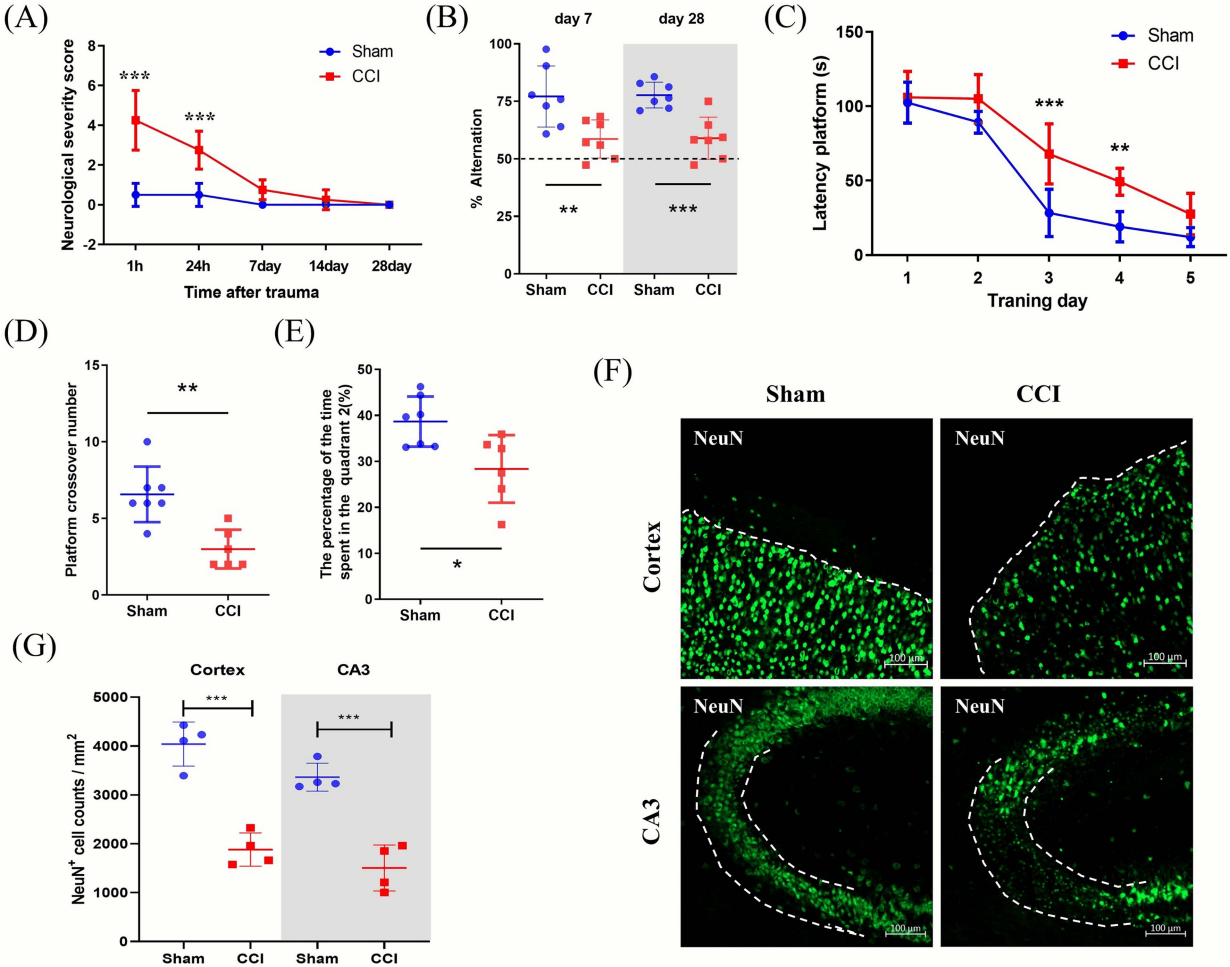
Controlled cortical impact (CCI) induces acute neurological deficits and chronic neuronal loss and cognitive impairment in mice. **(A)** Neurological severity score (NSS) at 1, 3, 7 and 14 days after CCI; n = 8–12 mice/group. **(B)** Y-maze spontaneous alternation at 1 and 4 weeks post-CCI, reflecting working memory; n = 8–12 mice/group. **(C)** Escape latency during the 5-day Morris water maze (MWM) acquisition phase performed at 4 weeks post-CCI; n = 8–12 mice/group. **(D)** Number of platform crossings in the MWM probe trial. **(E)** Percentage of time spent in the target quadrant in the MWM probe trial. **(F)** Representative immunofluorescence images of the peri-lesional cortex and hippocampal CA3 region at 30 days post-CCI, stained for NeuN (neurons) and DAPI (nuclei); the cortex and CA3 sub-region are outlined by a white dotted line. Scale bar, 100 μm. **(G)** Quantification of NeuN-positive cells in the cortex and CA3 region; n = 6 mice/group. Data are mean ± SD. *P < 0.05, **P < 0.01, ***P < 0.001 vs sham. Statistical analysis: two-way ANOVA with Šídák’s *post-hoc* test for **(A–C)**; unpaired two-tailed Student’s t-test for **(D, E, G)**. Antibody and reagent details are provided in Methods.

**Figure 2:**
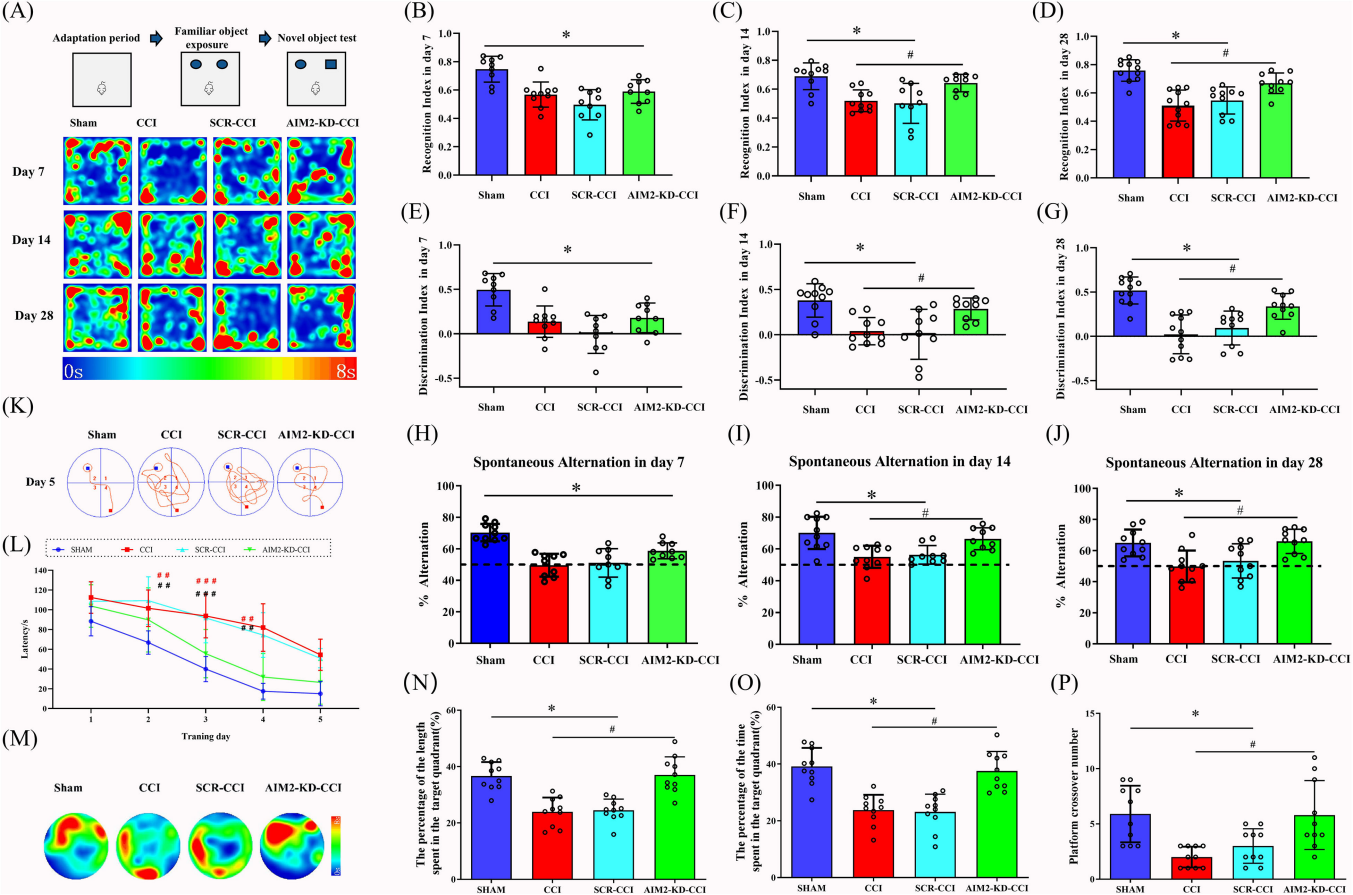
AAV-mediated neuronal AIM2 knockdown rescues hippocampus-dependent cognitive function after CCI. Cognitive function was assessed by novel object recognition (NOR), Y-Maze and MWM in mice that received CA3 stereotaxic injection of AAV-PhP.eB-shAIM2 or AAV-PhP.eB-shScr two weeks before CCI. **(A)** Representative heat maps from the NOR test at days 7, 14 and 28 post-CCI. **(B–D)** Recognition index from the NOR test. **(E–G)** Discrimination index from the NOR test. **(H–J)** Spontaneous alternation rate in the Y-Maze. **(K)** Representative swimming paths on day 5 of MWM acquisition training. **(L)** Escape latency over the 5-day MWM acquisition phase. **(M)** Representative heat maps from the MWM probe trial. **(N)** Percentage of total distance travelled in the target quadrant. **(O)** Percentage of total time spent in the target quadrant. **(P)** Number of platform crossings. Data are mean ± SD; n = 10 mice/group. *P < 0.05, **P < 0.01, ***P < 0.001 vs. Sham; #P < 0.05, ##P < 0.01, ###P < 0.001 vs. AAV-shScr-CCI. Statistical analysis: two-way ANOVA with Tukey’s *post hoc* test.

Y-Maze Spontaneous Alternation: Spatial working memory was assessed at 7, 14 and 28 days post-CCI, corresponding to the time points presented in **Figure 1B** (CCI characterization, 1 and 4 weeks) and **Figures 2H–J** (AAV-shAIM2 rescue, 14 and 28 days). Mice were placed in the center of a symmetrical Y-maze (arms: 30 cm long, 8 cm wide, 15 cm high; Shanghai XinRuan Information Technology Co., Ltd., model XR-XY1032) and allowed to explore freely for 8 min. Arm entries were recorded using video tracking software (EthoVision XT, Noldus). A spontaneous alternation was defined as consecutive entries into three different arms (i.e., A→B→C, B→C→A or C→A→B; an immediate return to the previously visited arm such as A→B→A is not counted). The percentage of alternation was calculated as: [(Number of alternations)/(Total number of arm entries – 2)] × 100%.

Novel Object Recognition (NOR): Recognition memory was assessed at 7, 14 and 28 days post-CCI, corresponding to the time points presented in **Figures 2A–G**. On day 1 (habituation), mice explored the empty arena (40 × 40 × 40 cm; Shanghai XinRuan Information Technology Co., Ltd., model XR-XX117) for 10 min. On day 2 (training), mice explored the arena containing two identical objects for 10 min. One hour later (testing), one familiar object was replaced with a novel object, and mice explored for 5 min. Exploration time (sniffing or touching the object with the nose) was recorded. The discrimination index (DI) was calculated as: [(Time exploring novel object) – (Time exploring familiar object)]/(Total exploration time) × 100%.

*Morris Water Maze (MWM)*: Spatial learning and memory were assessed starting on day 21 post-CCI over 5–7 consecutive days. The maze (Shanghai XinRuan Information Technology Co., Ltd., model XR-XM101) consisted of a circular pool (120 cm diameter) filled with opaque water (22 ± 1°C). A hidden platform (10 cm diameter) was submerged 1 cm below the water surface in a fixed quadrant. Mice underwent four training trials per day (60 or 90s maximum) starting from different quadrants. Escape latency (time to find the platform) was recorded using EthoVision XT. On the day after the last training day, a probe trial was conducted where the platform was removed, and mice swam freely for 60s. Time spent in the target quadrant and the number of platform location crossings were measured.

### Hippocampal neuron culture

Primary hippocampal neurons were prepared from neonatal mice on post-natal day 1 (P1), following established methods. Briefly, dissected hippocampi were enzymatically dissociated by incubation in 4 mL of 0.25% trypsin (Invitrogen) for 2 min at 37°C. The trypsin activity was quenched by the addition of 0.5 mL of fetal bovine serum (Gibco). A cell pellet was obtained by centrifuging the suspension at 900 g for 10 min. After resuspension in minimum essential medium (Gibco), the cells were plated onto 12.5 µg/mL poly-D-lysine-coated glass coverslips (12 mm × 12 mm) at a density of 2 × 10³ cells/mm². The neurons were maintained at 37°C in a 5% CO_2_ atmosphere in 2 mL of Neurobasal medium, which was freshly supplemented with 2 mM glutamine (Sigma) and 2% B-27 supplement (Gibco). A half-volume of the culture medium was replaced every three days. To minimize glial proliferation, the selective replication inhibitor arabinosylcytosine C (4 μM final concentration) was introduced to the cultures on day 3 for a 24-hour period. Subsequent experiments were performed on neuronal cultures at approximately day 8.

### Neuro-2a cell culture and differentiation

Mouse neuroblastoma Neuro-2a (N2A) cells (ATCC, CCL-131) were maintained in Dulbecco’s Modified Eagle Medium (DMEM, Gibco) supplemented with 10% fetal bovine serum (FBS, Gibco) and 1% penicillin/streptomycin (Gibco) at 37°C in a 5% CO_2_ incubator. For differentiation, cells were seeded onto poly-L-lysine coated plates/coverslips and cultured in reduced-serum medium (DMEM with 0.5% FBS) or serum-free medium (DMEM/F12 supplemented with B27 (Invitrogen, #17504044, 2% v/v) and potentially retinoic acid) for 48-72h. Differentiation was confirmed by morphology (neurite outgrowth) and immunofluorescence staining for βIII-tubulin (Tuj1) (Abcam, ab18207, 1:500) as described in the Immunofluorescence section.

### *In vitro* mechanical injury (scratch assay)

Primary hippocampal neurons were injured at DIV 7-10 (80-90% confluency, mature state with established networks, verified by β-III tubulin staining), and Neuro-2a cells at 70-80% confluency (log-phase growth). The injury method involved a 21-gauge needle drawn across the monolayer in a grid pattern (10x10 cross scratches per well, ~0.5 mm width), inducing consistent shear stress mimicking TBI mechanics; media was replaced post-injury to remove debris. This protocol, adapted from validated models, ensures reproducible cellular responses without excessive cell death (<20% baseline LDH at 0h). Control wells were treated identically but without scratching. For all *in vitro* assays, n=3–5 independent cultures per condition, with data as mean ± SEM and 95% CI where relevant.

### Immunofluorescence staining and confocal microscopy

At designated time points (24h or 28d post-CCI), mice were deeply anesthetized and transcardially perfused with ice-cold phosphate-buffered saline (PBS) followed by 4% paraformaldehyde (PFA) in PBS. Brains were post-fixed overnight in 4% PFA, cryoprotected in 30% sucrose/PBS, and sectioned coronally at 30 μm thickness using a cryostat (Leica CM1950 or similar). Free-floating sections were permeabilized and blocked (0.3% Triton X-100 and 5% normal goat serum (NGS) in PBS) for 1h at room temperature. Sections were then incubated overnight at 4°C with primary antibodies diluted in blocking buffer: anti-NeuN (CST, #12943, 1:1000), anti-Iba1 (GeneTex, GTX100042, 1:500), anti-GFAP (Cell Signaling Technology, #12389, 1:500), anti-AIM2 (Santa Cruz, sc-515424, 1:100), anti-ASC (Cell Signaling Technology, #67824, 1:800), anti-GSDMD (recognizes full-length and NT) (Affinity, AF4012, 1:100). After washing with PBS, sections were incubated for 1-2h at room temperature with appropriate Alexa Fluor-conjugated secondary antibodies (Cell Signaling Technology, Anti-mouse IgG (H+L), (Alexa Fluor^®^ 555 Conjugate), #4409, 1:1000 or Cell Signaling Technology, Anti-rat IgG (H+L), (Alexa Fluor^®^ 488 Conjugate), #4416, 1:1000). Nuclei were counterstained with DAPI (1 μg/mL). Sections were mounted onto slides using anti-fade mounting medium (e.g., ProLong Gold, Invitrogen). Cell counts: NeuN+ cells/mm² in predefined ROIs (3 sections/animal, n=3 mice/group, mean ± SEM, 95% CI).

**Figure 3:**
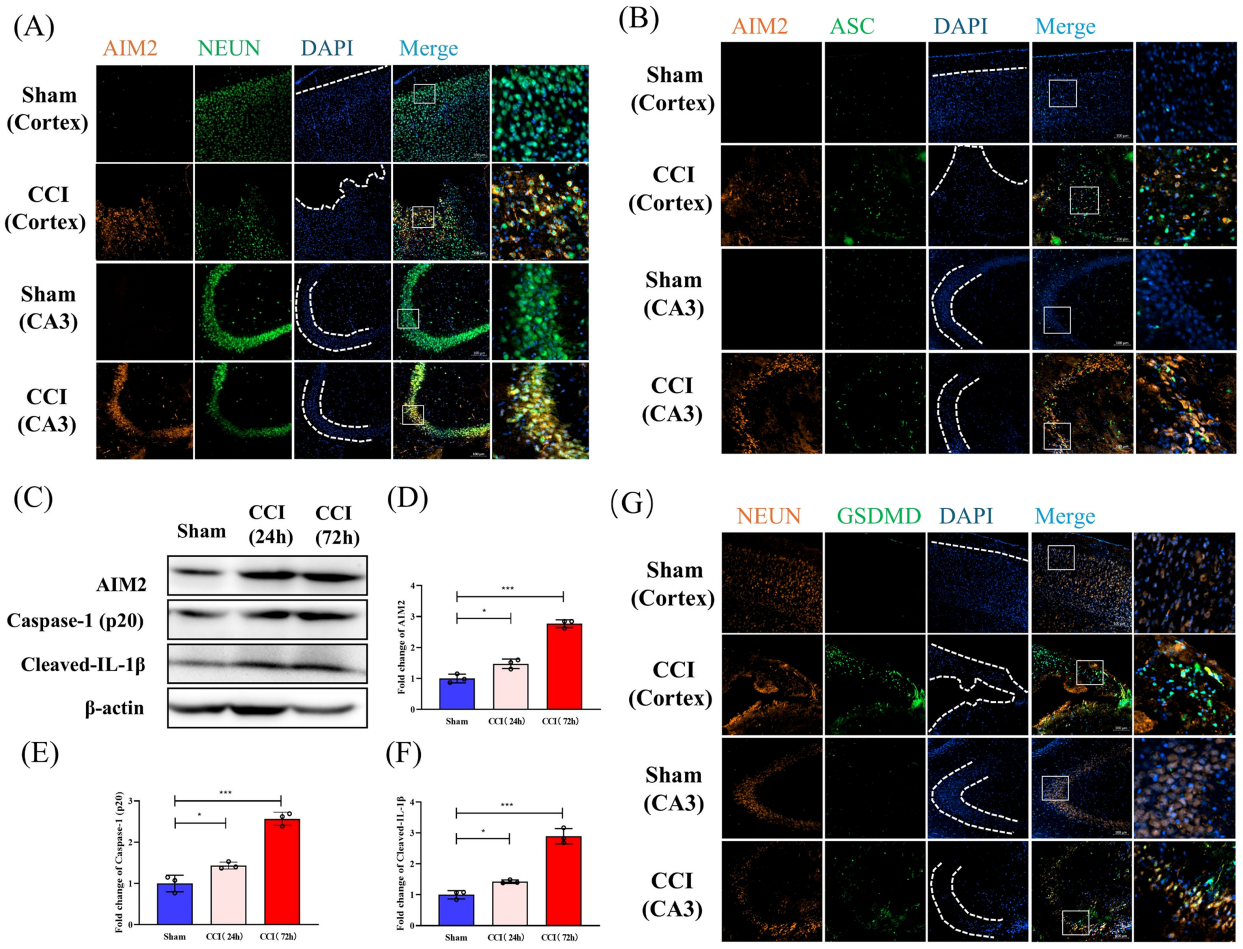
AIM2 and GSDMD co-localise with the neuronal marker NeuN in the peri-lesional cortex and hippocampal CA3 region 24 h after CCI. **(A)** Representative confocal images of AIM2 (green) and NeuN (red) in the peri-lesional cortex (top row) and CA3 region (bottom row) of Sham and CCI mice 24 h post-injury; nuclei counterstained with DAPI (blue). The CA3 sub-region is outlined by a white dotted line. Scale bar, 50 μm. **(B)** Quantification of AIM2 fluorescence intensity within NeuN-positive neurons. **(C, D)** Pearson’s correlation coefficient **(r)** and Mander’s overlap coefficients (M1, M2) for AIM2/NeuN co-localisation in cortex and CA3, computed by Costes’ regression-based automatic thresholding; numerical values: Cortex Sham r = 0.10, M1 = 0.20, M2 = 0.52; Cortex CCI r = 0.41, M1 = 0.99, M2 = 0.98; CA3 Sham r = −0.02, M1 = 0.00, M2 = 0.47; CA3 CCI r = 0.64, M1 = 1.00, M2 = 0.95 (see **Supplementary Figure 12** for bar-chart visualisation). **(E)** Representative Western blots of AIM2, ASC, cleaved caspase-1 (p20) and IL-1β in hippocampal lysates from Sham and CCI mice; β-actin served as loading control. **(F)** Quantification of corresponding protein levels normalised to β-actin. **(G)** Representative confocal images of GSDMD (green) and NeuN (red) in the peri-lesional cortex and CA3 region of Sham and CCI mice 24 h post-injury; nuclei counterstained with DAPI (blue). The CA3 sub-region is outlined by a white dotted line. Scale bar, 50 μm. **(H)** Pearson’s correlation coefficient (r) and Mander’s overlap coefficients (M1, M2) for GSDMD/NeuN co-localisation in cortex and CA3 (Costes’ thresholding): Cortex Sham r = 0.37, M1 = 0.97, M2 = 1.00; Cortex CCI r = 0.49, M1 = 1.00, M2 = 0.93; CA3 Sham r = 0.11, M1 = 0.65, M2 = 1.00; CA3 CCI r = 0.59, M1 = 0.99, M2 = 1.00 (see **Supplementary Figure 12**). Data are mean ± SEM from three independent biological replicates per group, with ≥ 3 technical replicates each. *P < 0.05, **P < 0.01, ***P < 0.001 versus Sham; unpaired two-tailed Student’s t-test. The original.czi files and the Python script used to compute the colocalisation coefficients are deposited as **Figure 3** source data with this submission.

**Figure 4:**
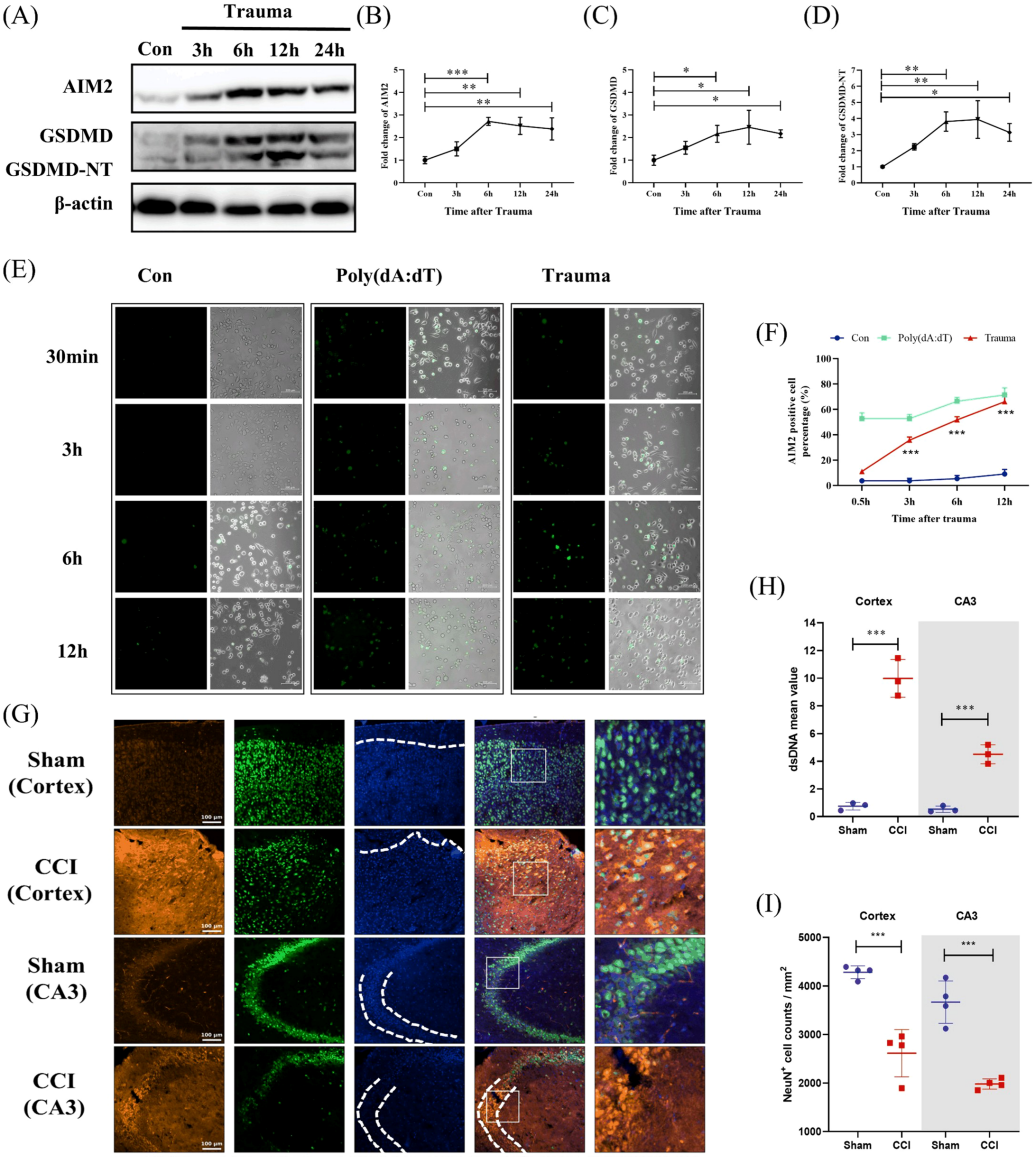
Mechanical injury triggers AIM2 inflammasome activation, neuronal pyroptosis and cytoplasmic dsDNA accumulation in injured neurons. **(A)** Representative Western blots of AIM2, ASC, cleaved caspase-1 (p20), full-length GSDMD and GSDMD N-terminal fragment (GSDMD-NT) in primary hippocampal neurons at 3, 6, 12 and 24 h after *in vitro* mechanical scratch injury; β-actin served as the loading control. **(B–D)** Densitometric quantification of AIM2, cleaved caspase-1 (p20) and GSDMD-NT normalised to β-actin across the post-injury time course. **(E)** LDH release into the culture supernatant at 3, 6 and 12 h post-injury, expressed as fold change relative to the untreated control. **(F)** ELISA quantification of IL-1β and IL-18 release into the culture supernatant 12 h post-injury. **(G)** Representative immunofluorescence images of double-stranded DNA (dsDNA, orange; Millipore MAB1293, clone AE-2), the neuronal marker NeuN (green) and Hoechst (blue) in the peri-lesional cortex (top row) and hippocampal CA3 region (bottom row) of Sham and CCI mice 24 h post-injury, showing accumulation of cytoplasmic dsDNA in injured neurons. Acquisition parameters (ZEISS LSM 800; ZEN 2.3 blue; Plan-Apochromat 10×/0.45 air; 488 nm 0.5%; 561 nm 1%; PMT 720–740 V; pinhole ≈ 1 AU; pixel dwell 1.52 μs; 8-bit, 512 × 512 px) were held constant across all groups. The CCI CA3 panel was horizontally flipped at the display stage to match the orientation of the Sham CA3 panel, with no effect on quantitative analysis. Linear, vessel-like signal in the red channel represents residual erythrocyte autofluorescence at 555–580 nm and was excluded from quantification. Scale bar, 50 μm. Data are mean ± SEM from three independent biological replicates, each with ≥ 3 technical replicates. *P < 0.05, **P < 0.01, ***P < 0.001 versus control or Sham; one-way ANOVA with Tukey’s *post-hoc* test. The original raw.czi files for all **Figure 4G** panels and the complete acquisition-parameter table are deposited as **Figure 4** source data with this submission.

**Figure 5:**
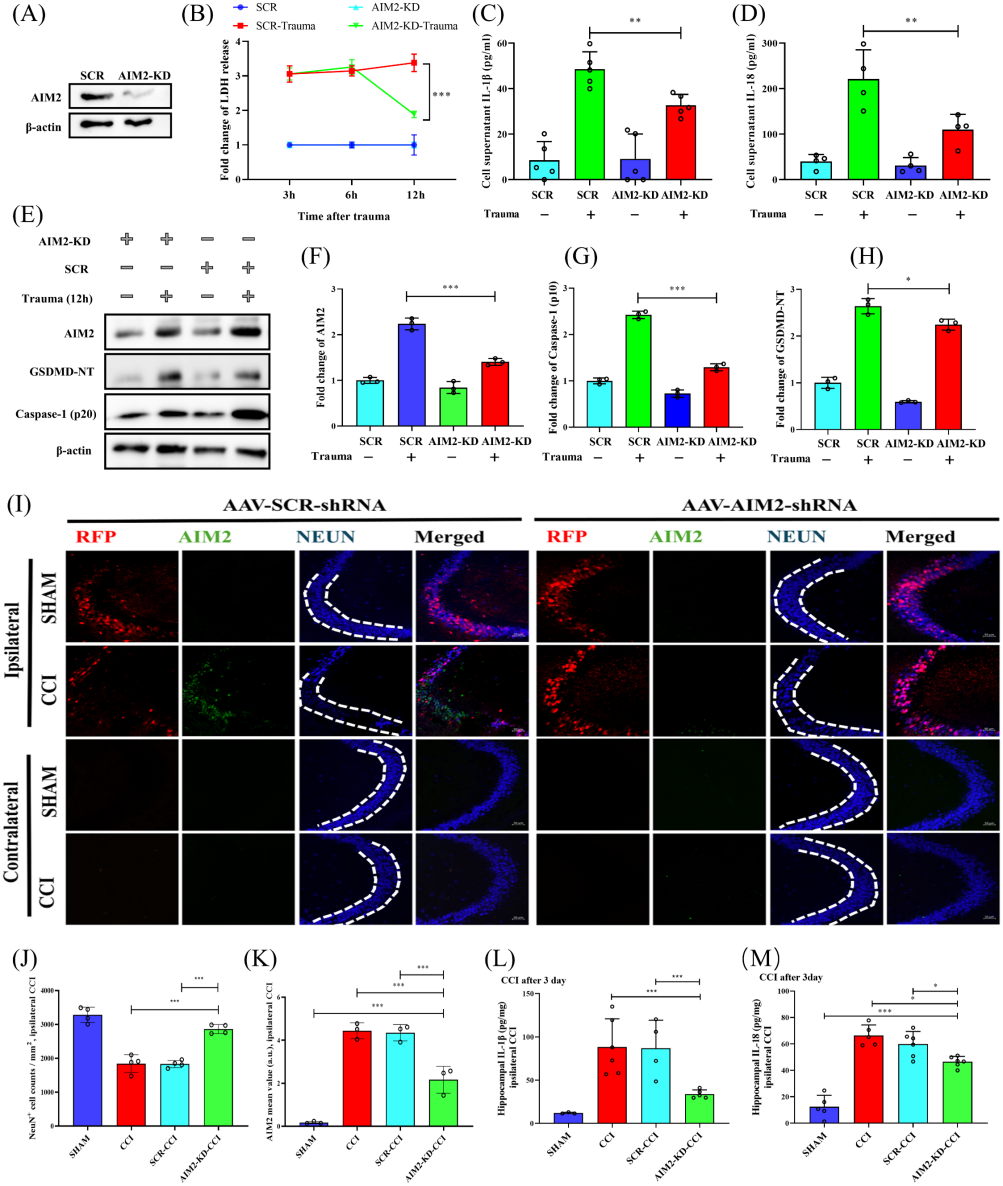
Neuron-targeted AIM2 knockdown attenuates pyroptosis and neuroinflammation *in vitro* and *in vivo*. **(A)** Western blot validating AIM2 knockdown efficiency by lentiviral AIM2 shRNA (AIM2-KD) in primary hippocampal neurons, compared with a scramble shRNA control (SCR). **(B)** LDH-release assay measuring cytotoxicity at 3, 6 and 12 h post-mechanical injury. **(C, D)** ELISA quantification of IL-1β and IL-18 release into the culture supernatant 12 h post-injury. **(E)** Representative Western blots of AIM2, cleaved caspase-1 (p10) and GSDMD-NT 12 h post-injury. **(F–H)** Quantification of corresponding protein levels, normalised to SCR. **(I)** Representative immunofluorescence images of the hippocampal CA3 region 28 days post-CCI from mice that received stereotaxic AAV-PhP.eB-shAIM2 or AAV-PhP.eB-shScr injection. Staining shows NeuN (red) and AIM2 (green); the CA3 sub-region is outlined by a white dotted line. AAVs were pre-injected ipsilaterally two weeks prior to CCI; RFP was used as the AAV transduction reporter. Scale bar, 100 μm. **(J)** Quantification of NeuN-positive cell density. **(K)** Quantification of AIM2 mean fluorescence intensity. **(L, M)** ELISA for IL-1β and IL-18 in hippocampal lysates 3 days post-CCI. Panels A–H, lentiviral shRNA knockdown *in vitro* (primary neurons); Panel I, AAV-PhP.eB-mediated shAIM2 *in vivo*. Data are mean ± SD from three independent experiments (*in vitro*) or n = 4–6 mice/group (*in vivo*). *P < 0.05, **P < 0.01, ***P < 0.001; pairwise comparisons are indicated. Statistical analysis: one-way ANOVA with Tukey’s *post hoc* test.

Confocal acquisition parameters. Confocal images of fixed brain sections (**Figures 1F**, **3A, B, G**, **4G**, **5I**) were acquired on a ZEISS LSM 800 confocal laser scanning microscope (Carl Zeiss AG, Oberkochen, Germany) equipped with diode lasers at 405, 488, 561 and 640 nm and two GaAsP photomultiplier (PMT) detectors, using ZEN 2.3 (blue edition) software. The objective used for each panel is indicated in the respective figure legend; **Figure 4G** was acquired with a Plan-Apochromat 10×/0.45 NA air objective (pixel size 1.25 μm), while high-magnification images for colocalization analysis (**Figures 3A, G**) were acquired with a Plan-Apochromat 63×/1.4 NA oil DIC objective. Acquisition settings followed Zeiss-recommended parameters for fluorescence imaging of fixed brain tissue: laser power was kept low (0.5% for the 488 nm channel and 1% for the 561 nm channel; ≤ 3% for the 405 nm channel), master detector gain was set in the 720–740 V range and adjusted so that the brightest signal in each comparison set filled approximately 50% of the PMT dynamic range without saturation, the confocal pinhole was set to ~1 Airy unit (AU) for the longest-wavelength channel and the other channels were matched to the same optical-section thickness, pixel dwell time was 1.52 μs, and images were acquired at 8-bit depth with a frame size of 512 × 512 pixels (**Figure 4G**; ZEN default Range Indicator was monitored to confirm absence of pixel saturation in the dominant signal). The acquisition parameters used for the four **Figure 4G** panels are listed in the source-data table accompanying this submission and are also embedded as XML metadata in the deposited raw.czi files (see Data availability). Prior to acquisition, all mice were transcardially perfused with ice-cold PBS to remove blood cells; however, residual erythrocyte autofluorescence in the AF555 (red) channel — identifiable by its linear, vessel-like morphology and presence in both Sham and CCI sections — was readily distinguishable from nuclear and cytoplasmic dsDNA signal and was excluded from quantification.

Colocalization quantification. Pearson’s correlation coefficient (r) and Mander’s overlap coefficients (M1, M2) were computed for the AIM2/NeuN (**Figure 3A**) and GSDMD/NeuN (**Figure 3G**) image pairs using Costes’ regression-based automatic thresholding (24), implemented in Python with the scikit-image library (v0.25) and validated against the Fiji Coloc2 plugin. The orthogonal regression slope between channels was computed by principal-axis analysis, and the intensity threshold was iteratively lowered along the regression line until the Pearson’s correlation in the sub-threshold (background) pixel population fell to ≤ 0; coefficients above the Costes-derived thresholds were then calculated as r, M1 and M2. Coefficients were computed on the representative confocal images presented in the corresponding figure panels (one representative confocal field per group, 512 × 512 pixels at 1.25 µm/pixel, acquired with the ZEISS LSM 800 settings described above). The full per-pixel calculation and the Costes-derived thresholds for each panel are deposited as **Figure 3** source data, together with the reproducible Python script.

TUNEL Assay: Apoptotic cell death was detected with the One Step TUNEL Apoptosis Assay Kit (Beyotime, C1088) according to the manufacturer’s instructions. Sections were co-stained with anti-NeuN to identify neuronal TUNEL-positive cells.

dsDNA Staining: To visualize cytoplasmic dsDNA, sections were incubated with an anti-dsDNA antibody (Millipore, MAB1293, 1:100) using the immunofluorescence protocol described above, and were co-stained with anti-NeuN for neuronal identification. Images were acquired with a confocal microscope (Zeiss LSM 800) using 20×, 40× or 63× oil objectives. For quantification of TUNEL-positive cells, Iba1-positive area, GFAP-positive area and NeuN-positive cell counts, images were obtained from standardized regions (e.g., the peri-lesional cortical layer or the CA3 pyramidal layer) and quantified in ImageJ by an investigator blinded to group assignment.

### Enzyme-linked immunosorbent assay

Hippocampal tissue from the ipsilateral hemisphere was rapidly dissected, weighed and homogenized in lysis buffer (RIPA buffer with protease inhibitors). Homogenates were centrifuged at 14,000 × g for 15 min at 4°C and supernatants were collected. Protein concentrations were measured with the BCA Protein Assay Kit (Thermo Fisher Scientific, #23225). Hippocampal IL-1β and IL-18 levels were quantified with commercial ELISA kits for mouse IL-1β (R&D Systems, MLB00C) and IL-18 (MBL, 7625) according to the manufacturers’ instructions. Absorbance was read at 450 nm with a microplate reader, and cytokine concentrations were normalized to total protein.

### Mitochondrial fractionation

Mitochondrial fractions were isolated by differential centrifugation. Briefly, homogenized tissue or cells were centrifuged at 800 × g (10 min, 4°C) to remove nuclei and debris; the supernatant was then centrifuged at 10,000 × g (15 min, 4°C) to pellet mitochondria. Fraction purity was confirmed by Western blot using compartment-specific markers: VDAC1 (mitochondria; Selleck F0314, 1:1000) and β-actin (cytosol; Sigma A5441, 1:5000), with cross-contamination of < 5% (quantified by densitometry, n = 3 replicates, mean ± SEM with 95% CI). For cultured cells the same protocol was applied to lysates prepared in mitochondrial isolation buffer.

### Cytosolic mtDNA quantification

Cytosolic fractions were obtained after the nuclear-pelleting spin and DNA was extracted with the QIAamp DNA Mini Kit (Qiagen). Gentle Dounce homogenisation was performed on ice in the presence of protease inhibitors to minimize mechanical disruption. Fraction purity was rigorously validated by Western blot using compartment-specific markers: β-actin (cytosol), VDAC1 (mitochondria) and Lamin B1 (nucleus), with cross-contamination of < 5% (densitometry). TERT was consistently undetectable in the cytosolic fraction, confirming the absence of nuclear contamination and ruling out non-specific mtDNA release during sample preparation. The targeted mtDNA genes (NADH/D-loop/non-NUMT) were quantified by qPCR against the nuclear control TERT, following Dang et al. (2017).

Delayed caspase-1 inhibition (VX-765 at 0 h versus 6 h post-injury). To test directly whether a caspase-1-dependent second wave operates downstream of AIM2, primary hippocampal neurons (DIV 7–10) were subjected to *in vitro* mechanical scratch injury and treated with VX-765 (20 μM; MedChemExpress, HY-13205) either concurrently with injury (0 h, whole-process inhibition) or with a 6-h delay (administered 6 h post-injury, after the caspase-8-dependent first wave of GSDMD-NT generation observed at 0.5–3 h). Cytosolic fractions were collected at 6, 9 and 12 h, and cytoplasmic mtDNA was quantified by qPCR using NADH normalized to nuclear TERT (NADH/TERT ratio; see ‘Cytosolic mtDNA quantification’ above). Culture supernatants were collected at 3, 6, 9 and 12 h, and LDH release was measured with the CytoTox 96 Non-Radioactive Cytotoxicity Assay (Promega, G1780) following the manufacturer’s instructions and expressed as fold change relative to the untreated control. Three independent biological replicates per group were performed, each with three or more technical replicates. Group comparisons used two-way ANOVA (treatment × time) with Tukey’s *post-hoc* test.

### Quantitative PCR

Total RNA was extracted with TRIzol reagent (Invitrogen). cDNA was synthesized with the HiScript II Reverse Transcriptase kit (Vazyme, R222). For mtDNA/nDNA quantification from cellular fractions or total DNA extracts (25), the protocol of Eric et al. was used. qPCR was performed with SYBR Green PCR Master Mix (Applied Biosystems, Foster City, CA, USA) on a QuantStudio 12K Flex (Applied Biosystems). Relative gene expression or DNA abundance was calculated by the 2^(−ΔΔCt) method. Primer sequences are listed in **Supplementary Table 1**.

### Western blot analysis

Hippocampal tissue (ipsilateral) or cultured cells were lysed in RIPA buffer (50 mM Tris-HCl pH 7.4, 150 mM NaCl, 1% NP-40, 0.5% sodium deoxycholate, 0.1% SDS) supplemented with protease and phosphatase inhibitor cocktails (Roche, #11836170001 and #4906837001). Protein concentrations were determined by BCA assay. Equal amounts of protein (20–40 μg per sample) were separated by SDS-PAGE on 10–15% gels and transferred to PVDF membranes (Millipore). Membranes were blocked with 5% non-fat dry milk or BSA in Tris-buffered saline with 0.1% Tween-20 (TBS-T) for 1 h at room temperature and then incubated overnight at 4°C with primary antibodies (full list and catalogue numbers in **Supplementary Table 2**). After three washes with TBS-T, membranes were incubated for 1 h at room temperature with HRP-conjugated secondary antibodies and developed by enhanced chemiluminescence (Bio-Rad ChemiDoc). Densitometric quantification was performed in ImageJ; β-actin or VDAC1 served as loading control for cytosolic or mitochondrial fractions, respectively.

### Cell viability and cytotoxicity assays

Lactate Dehydrogenase (LDH) Assay: Cell death was quantified by measuring LDH release into the culture supernatant at 3, 6, 12 and 24 h post-injury (matching the **Figure 5B** time-course) with the CytoTox 96^®^ Non-Radioactive Cytotoxicity Assay Kit (Promega, G1780) according to the manufacturer’s protocol. Absorbance was read at 490 nm.

Calcein-AM/Propidium Iodide (PI) Staining: Cell viability was assessed with the LIVE/DEAD™ Viability/Cytotoxicity Kit (Invitrogen, L3224). Cells were incubated with Calcein-AM (live cells, green) and PI (dead cells, red) for 15–30 min at 37°C and imaged by fluorescence microscopy.

### *In vitro* knockout and knockdown

Bax knockout (Bax-KO) and GSDMD knockout (GSDMD-KO) hippocampal neurons were generated separately using the SpCas9 system. Lentiviral vectors expressing SpCas9 together with single guide RNAs (sgRNAs) targeting mouse Bax (target sequence 5′-GCTGGACATTGGACTTCCTC-3′) or Gsdmd (5′-GCTACAGCCAGAAGACCTCG-3′) were constructed. A non-targeting sgRNA vector was used to generate control cells. Primary hippocampal neurons or Neuro-2a cells were transduced with lentiviral SpCas9/sgRNA (for GSDMD-KO or Bax-KO) or with shRNA (for AIM2 knockdown) at MOI 5–10, which was optimized by dose–response and yielded 70–90% transduction efficiency with > 85% viability (Trypan blue). Neurons were transduced at DIV 4–5 with an expression window of 48–72 h before injury. No antibiotic selection was applied, as the high transduction efficiency obtained without selection preserved neuronal viability (> 90% by MTT assay, n = 3–5 cultures). Knockout efficiency was confirmed at the genomic level by Sanger sequencing of PCR amplicons spanning the target site (to detect indels) and at the protein level by Western blot with anti-Bax and anti-GSDMD antibodies (**Supplementary Figures 4A, B**; > 70% reduction).

### Ethidium bromide-induced mtDNA depletion

To generate mtDNA-depleted (ρ^0^) Neuro-2a cells, parental cells were cultured for 8 days in standard growth medium supplemented with ethidium bromide (Sigma-Aldrich, E1510, 50 ng/mL), uridine (Sigma-Aldrich, U3003, 100 μg/mL) and sodium pyruvate (Gibco, 11360070, 1 mM). Complete mtDNA depletion was confirmed by qPCR comparing mitochondrial genome targets (NADH dehydrogenase subunit 1 [Nd1] and the D-loop) with nuclear genome targets (B2m and TERT).

### Bimolecular fluorescence complementation

To assess AIM2 oligomerization in live cells, Neuro-2a cells were co-transfected with plasmids expressing AIM2 fused to the non-fluorescent N-terminal (VN173) and C-terminal (VC155) fragments of the Venus fluorescent protein (pBiFC-VN173-AIM2 and pBiFC-VC155-AIM2 were constructed by seamless cloning). Transfections were performed with Lipofectamine 3000 (Invitrogen) according to the manufacturer’s instructions. At 24–48 h post-transfection, Venus fluorescence (indicating AIM2 oligomerization) was monitored by fluorescence microscopy. Poly(dA:dT) (Sigma-Aldrich, P0883, 1 μg/mL transfected for 4 h) served as a positive control for AIM2 activation.

### Mitochondrial membrane potential (ΔΨm) measurement

ΔΨm was assessed with JC-1 dye (Invitrogen, T3168). Cells were incubated with 2 μM JC-1 for 30 min at 37°C, washed, harvested and analyzed by immunofluorescence. JC-1 forms aggregates (red fluorescence, ≈ 590 nm emission) in healthy mitochondria with high ΔΨm and exists as monomers (green fluorescence, ≈ 530 nm emission) in the cytoplasm and in mitochondria with low ΔΨm. The red-to-green fluorescence intensity ratio was calculated per field and used as an index of ΔΨm. CCCP (10 μM, 30 min) served as a positive control for depolarization.

### Subcellular fractionation and mitochondrial protein localization

Mitochondrial and cytosolic fractions were isolated from cultured hippocampal neurons with a commercial kit (Mitochondria Isolation Kit for Cultured Cells, Thermo Fisher Scientific, #89874) according to the manufacturer’s instructions. Protein concentrations of each fraction were determined by BCA assay. Equal protein amounts were subjected to Western blot analysis as above, probing for GSDMD-NT, Bax and mitochondrial markers.

### Live-cell imaging

For live-cell visualization of GSDMD–mitochondria interaction, primary hippocampal neurons were transduced with a lentiviral vector encoding mCherry-tagged GSDMD; 24 h post-transduction, cells were co-stained with MitoTracker Green FM (Invitrogen, M7514, 100 nM, 30 min at 37°C) according to the manufacturer’s protocol. Live-cell time-lapse imaging was performed on a ZEISS LSM 880 confocal laser scanning microscope (Carl Zeiss AG, Oberkochen, Germany) equipped with a stage-top environmental chamber maintained at 37°C and 5% CO_2_ (humidified), using a Plan-Apochromat 63×/1.4 NA oil DIC objective and ZEN 2.3 (black edition) software. MitoTracker Green and mCherry were sequentially excited at 488 nm and 561 nm, respectively, and detected via two GaAsP PMTs with spectrally separated emission bands (490–550 nm and 580–650 nm). Acquisition parameters followed Zeiss-recommended settings for live-cell fluorescence imaging in order to minimize phototoxicity: laser power was kept low (0.5% for the 488 nm channel and 1% for the 561 nm channel), master gain was set in the 600–800 V range, confocal pinhole was 1 AU (set on the mCherry channel), pixel dwell time was 1.52 μs, frame size was 1024 × 1024 pixels at 16-bit depth, and z-step was set close to Nyquist sampling (~0.4 μm per slice). Images were captured at 0.5 h and 3 h after mechanical injury under identical settings in all conditions, and post-acquisition look-up tables, brightness/contrast and display thresholds were applied identically to all panels.

### Data analysis

Data are presented as mean ± standard error of the mean (SEM). Statistical analyses were performed in GraphPad Prism (version 9 or later). Normality was assessed by the Shapiro–Wilk test. For comparisons between two groups, an unpaired two-tailed Student’s t-test was used. For comparisons between three or more groups, one-way analysis of variance (ANOVA) followed by Tukey’s multiple-comparisons *post-hoc* test was used. For data analyzed at multiple time points, two-way ANOVA with Šídák’s multiple-comparisons *post-hoc* test was used. A P value of less than 0.05 was considered statistically significant. Statistical details (test, n, exact P value where applicable) are provided in each figure legend.

## Results

### Controlled cortical impact induces acute neurological deficits and persistent cognitive impairment

Traumatic brain injury triggers both immediate neurological dysfunction and long-term cognitive decline, but the temporal relationship between acute damage and persistent cognitive impairment remains poorly defined. Baseline neurological, motor and visual function were equivalent across groups before injury (**Supplementary Figure 8**). We first characterized the time course of TBI-related deficits in our CCI model. The CCI procedure produced immediate post-traumatic dysfunction, with a significant elevation of the Neurological Severity Score (NSS) at 24 h post-injury compared with sham animals (**Figure 1A**). Y-maze testing showed sustained working-memory impairment, with suppressed spontaneous alternation rates persisting from 1 to 4 weeks post-CCI (**Figure 1B**). Spatial-learning deficits persisted to 4 weeks post-CCI, with escape latency in the Morris water maze (MWM) significantly prolonged on training days 3 and 4 compared with sham (P < 0.01; **Figure 1C**). Chronic cognitive dysfunction was further confirmed at 28 days, with significant reductions in platform crossings and target-quadrant time compared with sham controls (**Figures 1D, E**).

Neuronal loss in the injured hemisphere paralleled the cognitive deficits, with significant reductions in NeuN-positive cells in both the cortex and hippocampal CA3 region (**Figures 1F, G**). Concurrent neuroinflammation was confirmed by activation and proliferation of microglia and reactive astrogliosis (**Supplementary Figures 1A, B**).

### Neuron-specific AIM2 inflammasome activation drives pyroptosis in acute TBI phase

Given the persistent neuroinflammation after TBI, we next asked whether AIM2 inflammasome activation contributes to the observed neuronal loss. Consistent with previous reports in stroke models (17, 26), we observed robust AIM2 inflammasome activation in cortical and hippocampal neurons at 24 h post-CCI, but not in other cell types (**Figure 3A**). AIM2/ASC colocalization (**Figure 3B**) and elevated AIM2, cleaved caspase-1 (p20) and cleaved IL-1β in ipsilateral brain homogenates (**Figures 3C–F**) at 24–72 h post-CCI further confirmed AIM2 inflammasome activation. We also detected clear colocalization of NeuN and GSDMD at 24 h post-CCI, indicating neuronal pyroptosis (**Figure 3G**). Quantitative colocalization analysis (Pearson’s r and Mander’s M1/M2; see **Supplementary Figure 12**; **Figure 3** legend) confirmed that, after CCI, AIM2 and GSDMD signals are predominantly located within NeuN-positive neurons (M1 ≈ 1.0 in injured cortex and CA3). Together, these findings highlight a neuron-enriched contribution of the AIM2 inflammasome to pyroptosis after TBI.

### Mechanical injury directly triggers AIM2 inflammasome activation in neurons

Primary injury in TBI is driven mainly by external mechanical forces, a critical but understudied activator of neuronal inflammasomes. To probe the causal relationship between mechanical injury and AIM2 inflammasome activation, we established an *in vitro* neuronal mechanical scratch (trauma) model. In primary mouse hippocampal neurons subjected to mechanical injury, AIM2, GSDMD-NT and GSDMD protein levels significantly increased at 6, 12 and 24 h post-trauma (P < 0.05–0.001, **Figures 4A–D**). In parallel, a bimolecular fluorescence complementation (BiFC) assay was used to directly visualize AIM2 multimerization in differentiated Neuro-2a (N2a) cells. Compared with the negative control, the positive-control group transfected with intracellular Poly(dA:dT) showed a high proportion of fluorescent protein-positive cells from 0.5 to 12 h post-transfection. The trauma group also showed time-dependent AIM2 oligomerization, with fluorescence-positive cells increasing from 10% (0.5 h) to 63% (12 h) post-injury (P < 0.001; **Figures 4E, F**), indicating progressive activation of the AIM2 inflammasome after mechanical injury. At 24 h post-trauma, intracellular dsDNA accumulation and its co-localization with NeuN in the peri-lesional cortex and hippocampal CA3 region were further confirmed (**Figures 4G–I**), strengthening the link between mechanical injury, cytosolic dsDNA accumulation and neuronal AIM2 activation.

### AAV-mediated AIM2 knockdown attenuates TBI-induced neuronal pyroptosis and cognitive dysfunction

We next assessed the therapeutic potential of targeting the AIM2 inflammasome in TBI-induced neuronal pyroptosis and cognitive dysfunction. Compared with neurons transduced with scrambled (Scr) shRNA lentivirus, neurons transduced with AIM2 shRNA lentivirus showed a significant reduction in AIM2 protein level (**Figure 5A**). In uninjured neurons, AIM2 knockdown did not significantly alter baseline LDH release (**Figure 5B**). In the *in vitro* mechanical injury model, shRNA-mediated AIM2 knockdown significantly reduced trauma-induced LDH release (P < 0.001, **Figure 5B**), as well as the release of IL-1β and IL-18 (P < 0.001, **Figures 5C, D**), and decreased the levels of AIM2, cleaved caspase-1 (p10) and GSDMD-NT (**Figures 5E–H**), indicating that AIM2 is necessary for mechanical-injury-induced neuronal pyroptosis.

Achieving targeted intervention *in vivo* requires precise modulation of inflammasome activity. Given the central role of the hippocampus in cognitive function and the observation of CCI-induced neuronal pyroptosis in the hippocampal CA3 region, we hypothesized that CCI may produce cognitive impairment in mice through neuronal pyroptosis in the hippocampus. We first validated AIM2-specific knockdown in mouse hippocampal CA3 neurons using stereotaxic intracerebral injection of an AAV vector carrying an AIM2-shRNA. The AIM2-shRNA effectively reduced AIM2 protein in the CA3 region at 24 h post-CCI (**Supplementary Figures 2A, B**). AAV vectors (AAV-shAIM2 or AAV-shScr) were stereotaxically injected into the hippocampal CA3 region two weeks before CCI to ensure optimal shRNA expression. Confocal imaging revealed strong colocalization between the AAV-encoded RFP reporter and NeuN, confirming that the vector predominantly transduced neurons in the ipsilateral hippocampus. At 28 days post-CCI, the AIM2-KD group showed a significant increase in the number of NeuN-positive neurons and a marked reduction in AIM2 expression in the CA3 region compared with both CCI and SCR-CCI controls (**Figures 5I–K**), demonstrating that targeted AIM2 knockdown effectively prevents CCI-induced neuronal loss in the hippocampus.

AIM2 knockdown also significantly attenuated the CCI-induced upregulation of IL-1β and IL-18 secretion in the hippocampus (**Figures 5L, M**). Together, these data indicate that targeted AIM2 knockdown in the hippocampus effectively rescues neuronal loss and suppresses pro-inflammatory cytokine release at the early stage after CCI.

Behaviorally, AAV-shAIM2 rescued CCI-induced deficits. In the Novel Object Recognition (NOR) task at 14 and 28 days post-injury, recognition and discrimination indices in AAV-shAIM2 mice were comparable to sham levels and significantly higher than in CCI and SCR-CCI controls (**Figures 2A–G**). The AIM2 knockdown group also showed a significantly higher alternation rate in the Y-maze at 14 and 28 days post-CCI compared with CCI and SCR-CCI controls and was indistinguishable from sham (**Figures 2H–J**). In the MWM at 28 days post-injury, the AIM2 knockdown group showed significantly reduced escape latency (**Figures 2K, L**) and improved path length, exploration time and platform-crossing counts (**Figures 2M–P**). Together, these behavioral data confirm that targeted AIM2 knockdown in hippocampal neurons effectively rescues TBI-induced cognitive dysfunction.

### Mitochondrial DNA leakage mediates mechanical injury-induced AIM2 inflammasome activation

We next examined the upstream triggers of AIM2 activation in mechanically injured neurons. Cytosolic dsDNA is a known AIM2 activator, but its origin after TBI has not been defined. After CCI, dsDNA accumulated specifically in the cytoplasm surrounding neuronal nuclei in the injured cortex and hippocampus, co-localizing with NeuN (**Figure 4G**). In the *in vitro* mechanical injury model, intracellular dsDNA accumulation was also detected in N2a cells at 24 h post-injury and co-localized with upregulated AIM2 (**Supplementary Figures 3A, B**). Although both nuclear and mitochondrial compartments contain abundant dsDNA, the mitochondrial origin of this cytoplasmic accumulation was supported by the absence of nuclear membrane disruption and the predominantly cytoplasmic, non-nuclear localization of the dsDNA signal.

**Figure 6:**
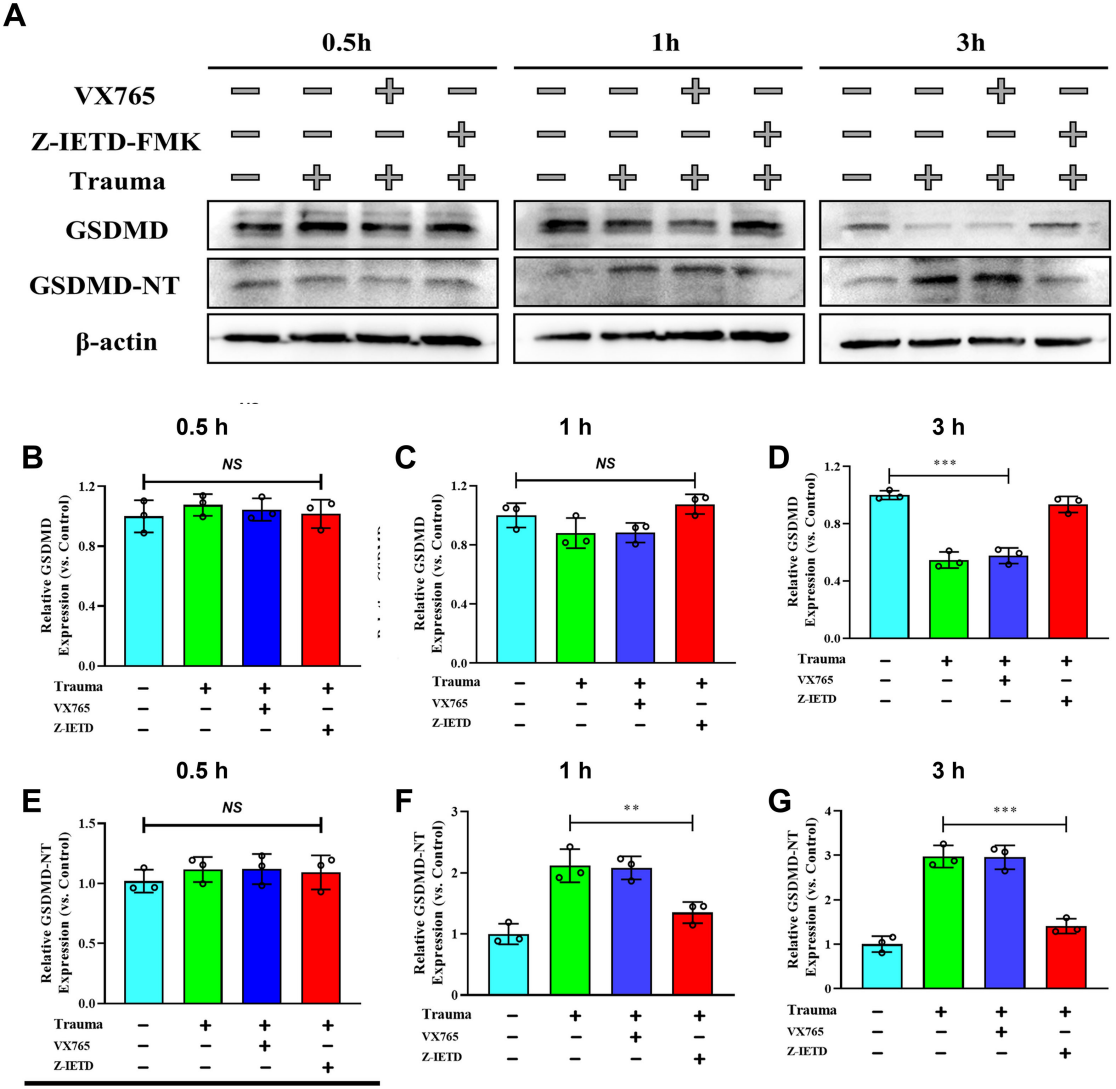
The caspase-8 inhibitor Z-IETD-FMK, but not the caspase-1 inhibitor VX-765, suppresses the early GSDMD cleavage event in mechanically injured primary hippocampal neurons. Primary mouse hippocampal neurons (DIV 7–10) were pre-treated for 30 min with either the caspase-8-selective inhibitor Z-IETD-FMK (20 μM) or the caspase-1-selective inhibitor VX-765 (20 μM) and then subjected to *in vitro* mechanical scratch injury. Cell lysates were harvested at 0.5, 1 and 3 h post-injury and analysed by Western blot for full-length GSDMD, GSDMD N-terminal fragment (GSDMD-NT) and β-actin (loading control). The +/− grid above each panel indicates the experimental condition for each lane: NC (no trauma, no inhibitor), Trauma alone, Trauma + VX-765 and Trauma + Z-IETD-FMK. **(A)** Representative Western blots from three independent biological replicates across all three time points. **(B–D)** Densitometric quantification of full-length GSDMD normalised to β-actin at 0.5 h **(B)**, 1 h **(C)** and 3 h **(D)**. Z-IETD-FMK restored full-length GSDMD to near-control levels by 3 h (***P < 0.001 versus Trauma), consistent with selective blockade of caspase-8-mediated cleavage; VX-765 had no significant effect at any time point (NS). **(E–G)** Densitometric quantification of GSDMD-NT normalised to β-actin at 0.5 h **(E)**, 1 h **(F)** and 3 h **(G)**. Z-IETD-FMK markedly reduced GSDMD-NT generation at 1 h (**P < 0.01 versus Trauma + VX-765) and 3 h (***P < 0.001 versus Trauma + VX-765), whereas VX-765 had no significant effect at any time point (NS versus Trauma). Data are mean ± SEM from n = 3 independent biological replicates per condition, each with ≥ 3 technical replicates; one-way ANOVA with Tukey’s *post-hoc* test. NS, not significant; **P < 0.01; ***P < 0.001.

**Figure 7:**
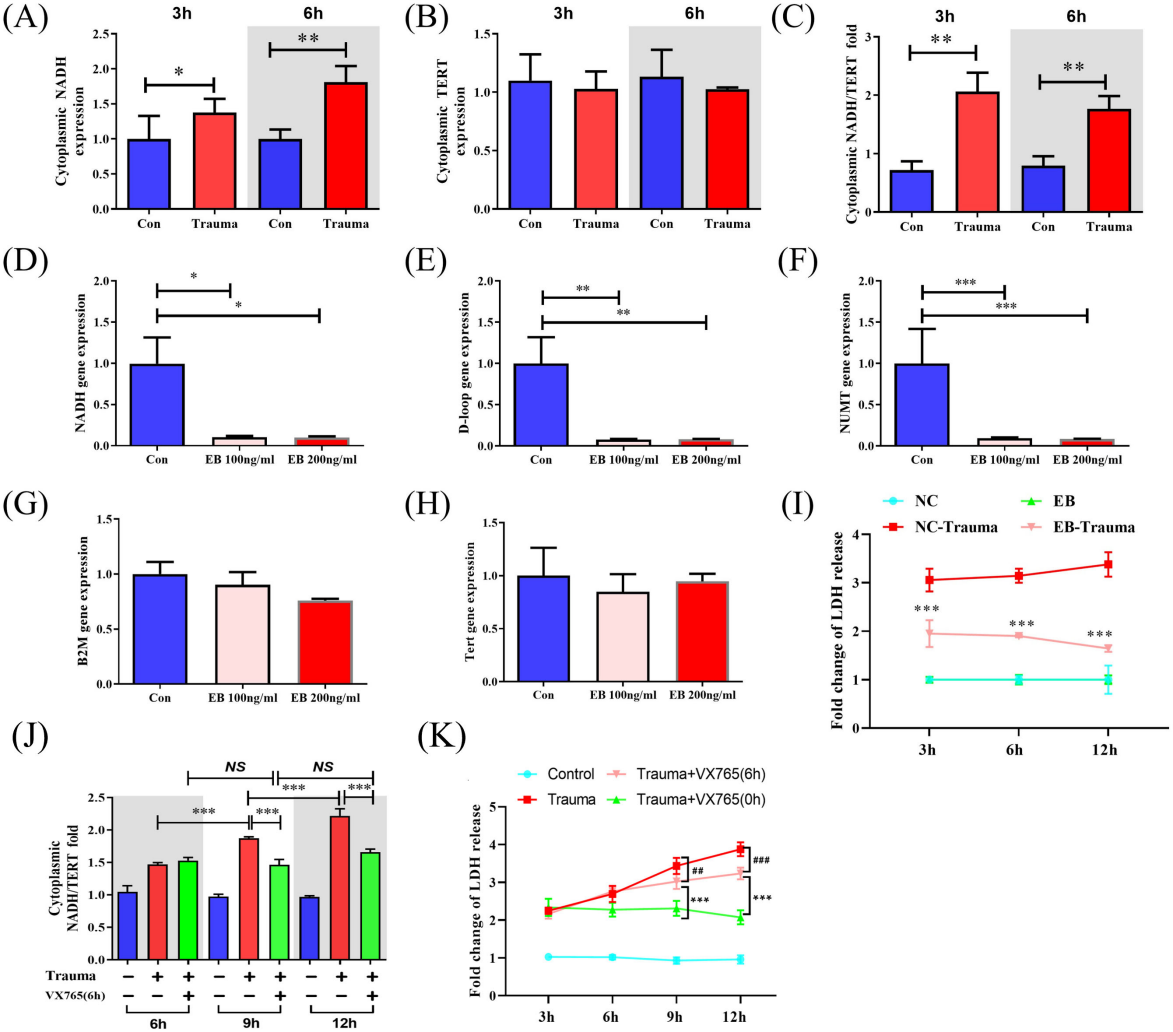
Cytosolic dsDNA originates from mitochondria; ethidium bromide-mediated mtDNA depletion attenuates neuronal pyroptosis; and delayed caspase-1 inhibition directly demonstrates the self-perpetuating second-wave amplification loop. **(A, B)** qPCR quantification of cytosolic dsDNA in primary hippocampal neurons after *in vitro* mechanical injury, using mitochondrial markers (mt-Nd1, D-loop) versus the nuclear control Tert; cell-fractionation purity was confirmed by Western blot of β-actin (cytosolic), VDAC1 (mitochondrial outer membrane), Lamin B1 (nuclear) and COX IV (mitochondrial inner membrane). **(C–F)** Ethidium bromide (EB) mtDNA depletion in primary neurons: dose response (50–200 ng/mL, 7-day pre-treatment), validated by qPCR for mt-Nd1, D-loop and NuMT versus nuclear Tert and B2M (~ 80% depletion at 200 ng/mL). **(G, H)** Nuclear control genes (B2M, Tert) were unaffected by EB, confirming mtDNA-specific depletion. **(I)** EB pre-treatment significantly attenuated injury-induced LDH release across the post-injury time course (3, 6 and 12 h), confirming mtDNA as the primary AIM2 activator. **(J)** [NEW] Direct experimental demonstration of a self-perpetuating second wave of mtDNA release. Primary hippocampal neurons were subjected to *in vitro* mechanical injury and treated with the caspase-1 inhibitor VX-765 (20 μM) administered 6 h post-injury — that is, after the first-wave (0.5–3 h) caspase-8-dependent GSDMD-NT generation (**Figure 6**) — thereby isolating the second, AIM2/caspase-1-driven amplification step. Cytoplasmic mtDNA was quantified by qPCR using cytosolic NADH normalised to nuclear TERT (NADH/TERT ratio). In Trauma-only cultures, cytoplasmic NADH/TERT rose progressively at 9 h and 12 h (~1.87- and ~2.21-fold versus Control), indicating a second wave of mtDNA release beyond the initial 3–6 h burst. Delayed VX-765 (green bars) significantly suppressed this late-phase second wave at both 9 h (***P < 0.001 versus Trauma) and 12 h (***P < 0.001 versus Trauma) without altering the 6 h value (NS), providing direct causal evidence that AIM2/caspase-1 activity downstream of the first wave drives the second, self-perpetuating wave of cytosolic mtDNA release. **(K)** [NEW] LDH release across the post-injury time course (3, 6, 9 and 12 h) in four groups: untreated control, Trauma alone, Trauma + VX-765 administered at 0 h (concurrent, whole-process inhibition) and Trauma + VX-765 administered at 6 h (delayed, second-wave-only inhibition). Concurrent VX-765 (0 h) almost completely abolished LDH release across the full time course (***P < 0.001 versus Trauma); critically, delayed VX-765 (6 h) still significantly attenuated LDH release at 9 h (##P < 0.01 versus Trauma) and 12 h (###P < 0.001 versus Trauma), defining a therapeutically tractable second-wave amplification window in which caspase-1 inhibition rescues neuronal viability even after the early caspase-8-driven first wave has occurred. Data are mean ± SEM from n = 3 independent biological replicates per group, each with three or more technical replicates. Statistical analysis: two-way ANOVA (treatment × time) with Tukey’s *post-hoc* test; NS, not significant; **/##P < 0.01; ***/###P < 0.001. Orthogonal pharmacological mtDNA depletion using the POLG-selective inhibitor 2′,3′-dideoxycytidine (ddC) is provided as **Supplementary Figure 7**.

Delayed caspase-1 inhibition provides direct evidence for a self-perpetuating second wave of mtDNA release. Anticipating our subsequent caspase-8-selective inhibitor data (presented below as **Figure 6**), in which the early (0.5–3 h) GSDMD-NT generation is shown to be caspase-1-independent, administering the caspase-1 inhibitor VX-765 after this first wave (6 h post-injury) allowed us to interrogate the secondary, AIM2/caspase-1-driven amplification step in isolation. In Trauma-only cultures, cytoplasmic NADH/TERT continued to rise beyond the initial 3–6 h burst, reaching ~1.87-fold of control at 9 h and ~2.21-fold at 12 h (**Figure 7J**). Delayed VX-765 (20 μM applied at 6 h) significantly suppressed this late-phase second wave at both 9 h and 12 h (***P < 0.001 versus Trauma) without altering the 6 h value (NS), providing direct causal evidence that caspase-1 activity downstream of AIM2 drives a discrete, self-perpetuating second wave of cytosolic mtDNA release. In parallel, concurrent VX-765 (0 h, whole-process inhibition) virtually abolished injury-induced LDH release across the entire post-injury time course (3, 6, 9 and 12 h; ***P < 0.001 versus Trauma); critically, delayed VX-765 still significantly attenuated LDH release at the late time points (9 h, ##P < 0.01; 12 h, ###P < 0.001 versus Trauma) (**Figure 7K**), defining a therapeutically tractable second-wave window in which caspase-1 inhibition can rescue neuronal viability even after the early caspase-8-driven first wave. Together, these data formally close the self-perpetuating amplification loop within the GSDMD–mtDNA–AIM2 axis (n = 3 independent biological replicates per group, each with ≥ 3 technical replicates).

Delayed caspase-1 inhibition directly demonstrates a self-perpetuating second wave of mtDNA release. Because our caspase-8–selective inhibitor experiments (**Figure 6**) showed that the early (0.5–3 h) GSDMD-NT generation is caspase-1–independent, administering VX-765 after this first wave (at 6 h post-injury) allowed us to selectively interrogate the secondary, AIM2/caspase-1–driven amplification step. In primary hippocampal neurons, Trauma alone caused a progressive rise in cytoplasmic NADH/TERT that extended well beyond the initial 3–6 h burst, reaching ~1.87-fold of control at 9 h and ~2.21-fold at 12 h (**Figure 7J**). Delayed VX-765 administration (20 μM at 6 h) significantly suppressed this late-phase second wave at both 9 h and 12 h (***P < 0.001 vs. Trauma), without altering the 6 h value (NS), providing direct causal evidence that AIM2/caspase-1 activity downstream of the first wave drives a second, self-perpetuating wave of cytoplasmic mtDNA release. Concurrent VX-765 administration (0 h, full-process inhibition) almost completely prevented injury-induced LDH release across the entire post-injury time course (3, 6, 9 and 12 h; ***P < 0.001 vs. Trauma), and critically, delayed VX-765 (6 h) still significantly attenuated LDH release at the late time points (9 h, ##P < 0.01; 12 h, ###P < 0.001 vs. Trauma) (**Figure 7K**). Together, these data formally close the self-perpetuating amplification loop within the GSDMD–mtDNA–AIM2 axis and define a therapeutically tractable second-wave window in which caspase-1 inhibition can still salvage neuronal viability even after the early caspase-8–driven first wave has occurred (n = 3 independent biological replicates per group, ≥ 3 technical replicates each).

### GSDMD pores disrupt mitochondrial membrane potential and promote mtDNA leakage

**Figure 8:**
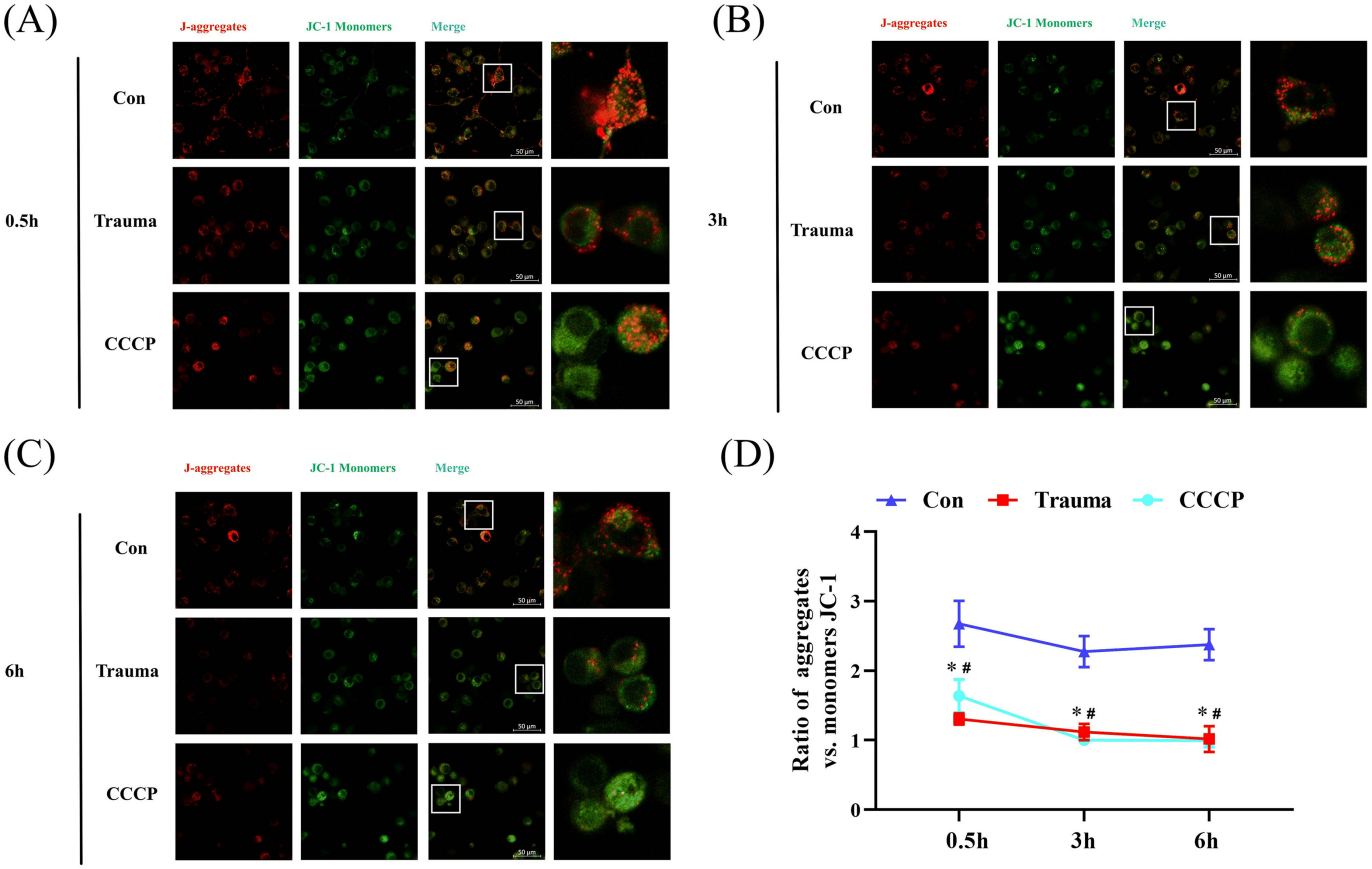
Mechanical injury induces a rapid loss of mitochondrial membrane potential (ΔΨm) in primary hippocampal neurons. **(A–C)** Representative fluorescence images of JC-1-stained hippocampal neurons at 0.5, 3 and 6 h after mechanical injury. CCCP (10 µM) served as a positive control for depolarisation. Red fluorescence (JC-1 aggregates) indicates high ΔΨm; green fluorescence (JC-1 monomers) indicates low ΔΨm. Scale bar, 50 μm. **(D)** Quantification of the red/green fluorescence intensity ratio as a proxy for ΔΨm over time. Data are mean ± SEM from three independent experiments. *P < 0.05 vs. Con; #P < 0.05 vs. corresponding time-matched Con. Statistical analysis: two-way ANOVA with Šídák’s *post hoc* test.

To dissect how mtDNA leakage occurs, we examined mitochondrial integrity after injury. Mitochondrial membrane permeabilization is a critical step in mtDNA release. JC-1 staining showed that mechanical injury and CCCP (an oxidative-phosphorylation uncoupler used as a positive control for ΔΨm loss) significantly reduced mitochondrial membrane potential (ΔΨm) compared with untreated controls at 0.5 h (**Figure 8A**); ΔΨm loss in both the injury and CCCP groups progressively worsened at 3 and 6 h (**Figures 8B–D**).

**Figure 9:**
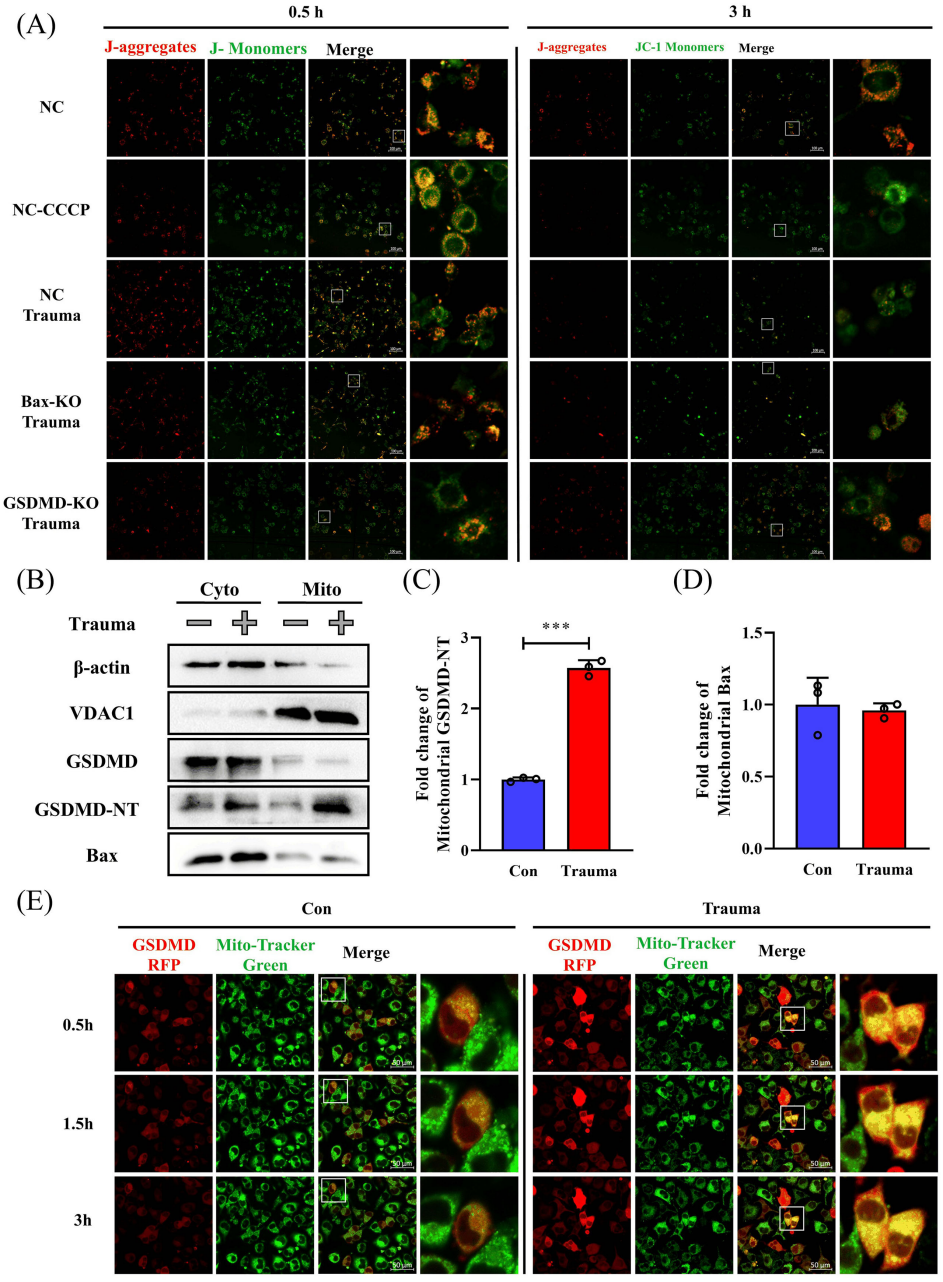
GSDMD, but not Bax, mediates early mitochondrial damage and mtDNA release after mechanical injury. **(A)** Quantification of mitochondrial membrane potential (ΔΨm) by JC-1 in Bax-KO, GSDMD-KO and control hippocampal neurons at 0.5 and 3 h post-injury. KO neurons were generated by lentiviral CRISPR/Cas9 (see Methods, ‘*In vitro* knockout and knockdown’). **(B)** Western blot of GSDMD, GSDMD-NT and Bax in cytosolic and mitochondrial fractions 3 h post-injury; β-actin and VDAC1 served as cytosolic and mitochondrial loading controls, respectively. **(C, D)** Densitometric quantification of mitochondrial GSDMD-NT and Bax, normalised to VDAC1. **(E)** Representative live-cell confocal images of neurons co-expressing mCherry-GSDMD (red) and stained with MitoTracker Green (green) at 3 h post-injury, illustrating GSDMD translocation to mitochondria. Scale bar, 50 μm. **(F, G)** Cytosolic mtDNA accumulation (mt-Nd1/Tert and D-loop/Tert ratios) in GSDMD-KO versus control neurons at 6 h post-injury, demonstrating that GSDMD ablation prevents injury-induced cytosolic mtDNA accumulation. Data are mean ± SEM from three independent biological replicates per group, each with ≥ 3 technical replicates. **P < 0.01, ***P < 0.001 versus the indicated comparator; one-way ANOVA with Tukey’s *post-hoc* test.

We next examined the molecular pathway responsible for mtDNA release after mechanical injury. During canonical apoptosis, Bax and Bak oligomerize to form pores in the outer mitochondrial membrane and facilitate mtDNA release (27). We therefore generated Bax-knockout (Bax-KO) neurons using a CRISPR-SpCas9 lentiviral system, with non-targeting sgRNA neurons as controls (**Supplementary Figure 4A**). JC-1 staining showed that, compared with control cells, Bax-KO did not inhibit or delay the mechanical-injury-induced ΔΨm loss at 0.5 and 3 h post-injury (**Figure 9A**). Bax/Bak channel-mediated mitochondrial permeabilization therefore does not appear to be the primary pathway for mtDNA release after mechanical injury. Consistent with this, confocal imaging confirmed that GSDMD progressively co-localizes with the mitochondrial marker TOM70 at 3 h before redistributing to the plasma membrane at 12 h (**Supplementary Figure 6**). Given that GSDMD-NT can permeabilize mitochondrial membranes and promote mtDNA release (14, 19, 20), we generated GSDMD-knockout (GSDMD-KO) cells with the same CRISPR-SpCas9 system (**Supplementary Figure 4B**). JC-1 staining showed that GSDMD-KO, unlike Bax-KO, significantly attenuated the injury-induced ΔΨm loss at 0.5 and 3 h (**Figure 9A**). Subcellular fractionation confirmed translocation of GSDMD-NT to the mitochondrial compartment within 3 h of injury, with mitochondrial GSDMD-NT levels significantly elevated compared with cytosolic levels (**Figures 9B–D**). Live-cell imaging of neurons co-expressing mCherry-GSDMD and stained with MitoTracker Green further demonstrated GSDMD translocation to mitochondria at 3 h post-injury (**Figure 9E**). Together, these data identify GSDMD, rather than Bax, as the upstream effector responsible for early mitochondrial permeabilization and mtDNA release in mechanically injured neurons.

Mechanical injury rapidly triggers initial GSDMD cleavage in neurons via caspase-8, independently of canonical inflammasome caspase-1. To identify the upstream protease responsible for the initial proteolytic activation of GSDMD after mechanical injury (**Figures 4A–D**, **9B–D**), we pre-treated primary hippocampal neurons with the caspase-8-selective inhibitor Z-IETD-FMK (20 μM, 30 min before injury) or the caspase-1-selective inhibitor VX-765 (20 μM, 30 min before injury) and quantified GSDMD-NT levels by Western blot at 0.5, 1 and 3 h post-injury. Trauma alone induced a rapid rise in GSDMD-NT that was detectable from 0.5 h and sustained through 3 h, consistent with our earlier time course (**Figures 4A–D**). This early rise was markedly suppressed by Z-IETD-FMK but not by VX-765 at all three time points (**Figure 6A**). Densitometric quantification across three independent biological replicates (n = 3) confirmed the temporal selectivity: Z-IETD-FMK restored full-length GSDMD to near-control levels by 3 h (***P < 0.001 versus Trauma) and reduced GSDMD-NT to ~1.4-fold of control at 1 h (**P < 0.01) and 3 h (***P < 0.001), whereas VX-765 had no significant effect on either full-length GSDMD or GSDMD-NT at any time point (NS) (**Figures 6B–G**). β-actin loading controls were comparable across all lanes and full-length GSDMD levels were preserved, indicating that Z-IETD-FMK selectively blocked GSDMD cleavage rather than altering GSDMD expression. These data establish caspase-8, rather than caspase-1, as the upstream protease responsible for the initial GSDMD cleavage event after mechanical injury — consistent with previous reports of caspase-8-dependent GSDMD cleavage in non-canonical pyroptosis contexts (28). Together with our finding that caspase-1 activation occurs downstream of mtDNA–AIM2 inflammasome assembly (**Figure 5**), these results delineate a sequential proteolytic cascade in which caspase-8 initiates GSDMD cleavage to launch the self-perpetuating amplification loop, while caspase-1 subsequently sustains and amplifies the loop — as now directly demonstrated by delayed VX-765 administration (**Figures 7J, K**).

In summary, our findings define a mtDNA–AIM2 inflammasome–GSDMD axis as a neuron-intrinsic driver of TBI-related pyroptosis and provide a mechanistic basis for neuron-targeted neuroprotective strategies for TBI-CD.

**Figure 10:**
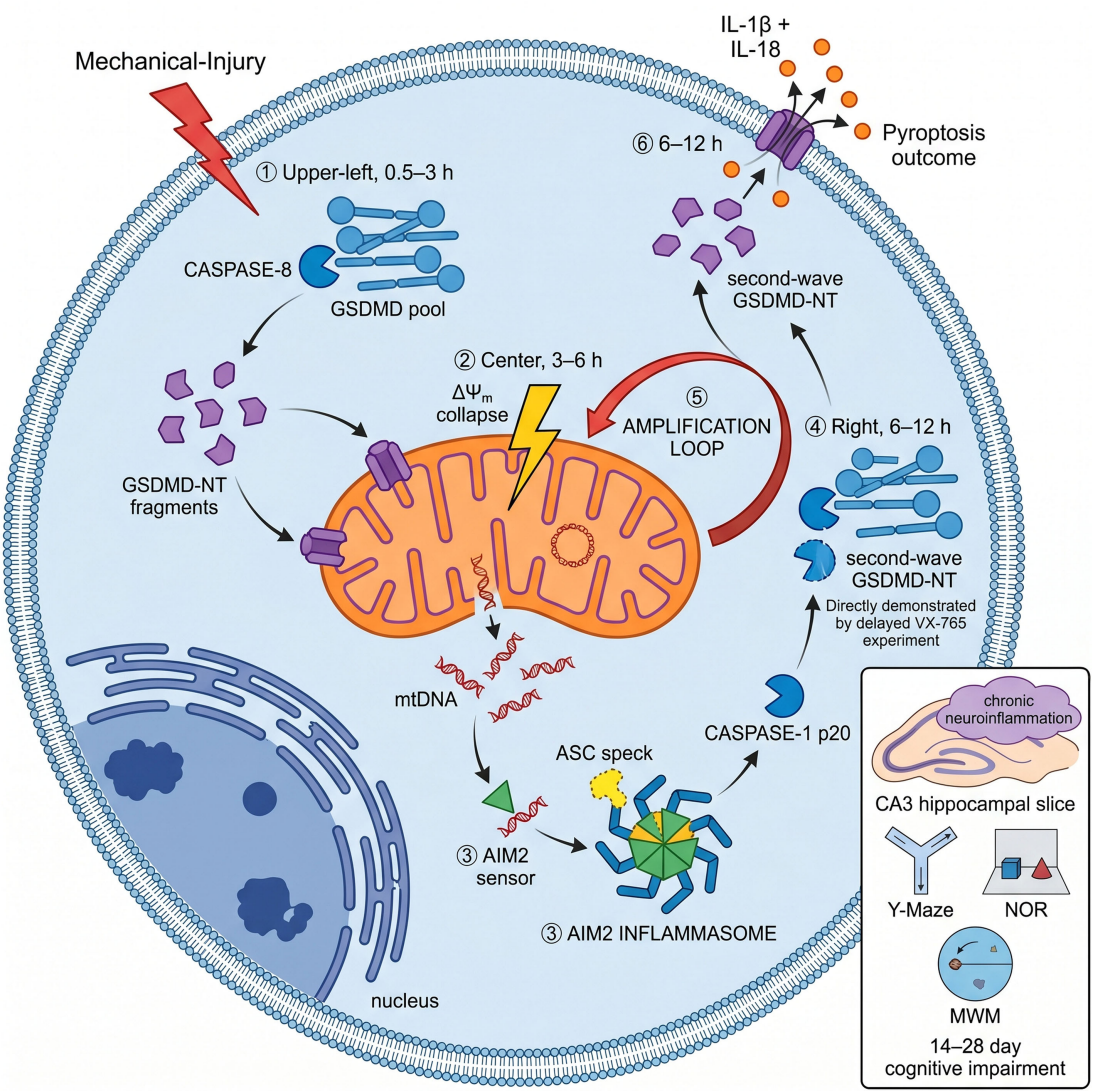
Proposed model of a neuron-intrinsic GSDMD–mtDNA–AIM2 inflammasome axis driving pyroptosis and cognitive decline after TBI, with a self-perpetuating amplification loop. Mechanical injury rapidly activates caspase-8, which cleaves GSDMD to generate GSDMD-NT (0.5–3 h). GSDMD-NT inserts into the mitochondrial outer membrane, dissipates ΔΨm and triggers cytosolic mtDNA release (3–6 h). Cytosolic mtDNA then licences the neuronal AIM2 inflammasome, leading to ASC speck formation and caspase-1 activation, which cleaves additional GSDMD and amplifies GSDMD-NT generation. Amplified GSDMD-NT further permeabilises the mitochondrial membrane (sustaining mtDNA release; second wave) and forms pores in the plasma membrane to execute pyroptosis. The resulting neuronal loss and DAMP release in the hippocampal CA3 region propagate chronic neuroinflammation and long-term cognitive impairment at 14–28 days post-CCI. Solid arrows indicate experimentally established steps; the second-wave amplification step (AIM2/caspase-1–derived GSDMD-NT re-targeting mitochondria) is now directly supported by delayed caspase-1 inhibition (**Figures 7J, K**). Traumatic brain injury (TBI) initiates a self-perpetuating amplification cycle within neurons: (1) mechanical injury triggers initial mitochondrial perturbation and caspase-8-mediated GSDMD cleavage (GSDMD-NT formation at 0.5–3 h; **Figure 6**); (2) GSDMD-NT permeabilises mitochondria, releasing mtDNA into the cytoplasm (3–6 h; **Figures 7A–C**); (3) cytosolic mtDNA activates the neuronal AIM2 inflammasome; (4) AIM2 recruits ASC and caspase-1, generating additional GSDMD cleavage; (5) caspase-1-derived GSDMD-NT re-targets mitochondria to drive a second, late-phase wave of mtDNA release at 6–12 h, now directly demonstrated by delayed VX-765 administration at 6 h post-injury (**Figures 7J, K**); (6) the resulting neuronal loss and DAMP release in the hippocampal CA3 region propagate chronic inflammation and TBI-induced cognitive dysfunction (14–28 days). This sustained, self-perpetuating cycle of neuronal pyroptosis, particularly in the hippocampus, drives neuronal loss and contributes substantially to the development of chronic cognitive dysfunction after TBI.

This study defines a neuron-intrinsic pathway that links the mechanical forces of traumatic brain injury (TBI) to sustained neuroinflammation and cognitive impairment. We show that, in the acute phase of TBI, mechanical injury directly triggers mitochondrial DNA (mtDNA) leakage within neurons, activating the AIM2 inflammasome and driving neuronal pyroptosis. We further identify the GSDMD N-terminal fragment (GSDMD-NT) as a participant in mitochondrial damage upstream of mtDNA release, establishing GSDMD-NT-mediated mitochondrial permeabilization as a critical step in the self-perpetuating amplification loop. Our proposed mechanistic model is summarized graphically in **Figure 10**. Therapeutically, targeted AAV-mediated AIM2 knockdown in the hippocampal CA3 region significantly attenuates neuronal pyroptosis and cognitive deficits in mice, identifying AIM2 as a candidate target for neuroprotective intervention after TBI.

## Discussion

Our data demonstrate a self-perpetuating amplification loop within the GSDMD–mtDNA–AIM2 pathway, now directly supported by delayed caspase-1 inhibition (**Figures 7J, K**). Early translocation of GSDMD-NT (0.5–3 h) disrupts mitochondrial integrity (reduced ΔΨm), facilitates mtDNA leakage (3–6 h) and activates AIM2 and caspase-1. Activated caspase-1 in turn generates additional GSDMD cleavage; the resulting GSDMD-NT inserts into the mitochondrial membrane and drives a second wave of mtDNA release (6–12 h), formally demonstrated here by delayed VX-765 administration (**Figures 7J, K**). In parallel, GSDMD-NT inserts into the plasma membrane to execute pyroptosis. GSDMD-KO — but not Bax-KO — breaks this cycle by preventing the initial mitochondrial dysfunction, consistent with reports that GSDMD-NT has direct pore-forming activity at mitochondrial membranes and accelerates pyroptosis.

Our data demonstrate a self-perpetuating amplification loop within the GSDMD–mtDNA–AIM2 pathway, now directly supported by delayed caspase-1 inhibition (**Figures 7J, K**). Early translocation of GSDMD-NT (0.5–3 h) disrupts mitochondrial integrity (reduced ΔΨm), facilitating mtDNA leakage (3–6 h) and activating AIM2 and caspase-1. Activated caspase-1 in turn generates additional GSDMD cleavage, producing more GSDMD-NT that inserts into the mitochondrial membrane and promotes further mtDNA release. In parallel, GSDMD-NT inserts into the plasma membrane to drive pyroptosis. GSDMD-KO — but not Bax-KO — breaks this cycle by preventing the initial mitochondrial dysfunction, consistent with reports that GSDMD-NT has direct pore-forming activity on mitochondrial membranes and accelerates pyroptosis.

The cytosolic mtDNA accumulation we measured by gentle fractionation and qPCR (**Figures 7A–C**) is corroborated functionally by the JC-1 ΔΨm collapse (**Figure 8**) and biochemically by the GSDMD-NT translocation to mitochondria documented at 0.5–3 h post-injury (**Figures 9B–D**) and by live-cell imaging (**Figure 9E**), all of which precede the cytosolic mtDNA peak at 3–6 h (**Figures 7A–C**). Together with the genetic GSDMD-KO/Bax-KO rescue data, the orthogonal pharmacological mtDNA depletion (EB in **Figure 7** and ddC in **Supplementary Figure 7**) and the delayed VX-765 second-wave evidence (**Figures 7J, K**), the mitochondrial dimension of the model is supported by a multi-layered characterization combining functional, biochemical, genetic and pharmacological readouts.

The loop initiates with mechanical injury producing early GSDMD activation and cleavage into GSDMD-NT, which translocates to mitochondria. Our *in vitro* mechanical-injury model shows activated GSDMD-NT in mitochondrial fractions within 0.5–3 h post-injury (**Figures 9B–D**), disrupting ΔΨm (JC-1 staining) and facilitating mtDNA leakage. Genetic knockout of GSDMD, but not Bax, prevents this injury-induced ΔΨm loss, ruling out classical apoptosis pathways such as Bax-mediated mitochondrial outer-membrane permeabilization (MOMP) and confirming a specific upstream role for GSDMD in mitochondrial damage. This is consistent with recent reports, including Miao et al. (2023), who demonstrated direct pore-forming activity of GSDMD-NT on mitochondrial membranes and a corresponding acceleration of pyroptosis.

Released mtDNA in turn serves as a ligand for AIM2, sustaining the loop through inflammasome activation. Our qPCR data reveal selective cytosolic mtDNA (but not nuclear DNA) elevation 3–6 h post-injury *in vitro*, directly correlating with AIM2 inflammasome activation (elevated AIM2, cleaved caspase-1, and GSDMD-NT in neurons; **Figures 3C–F**). Depletion of mtDNA with ethidium bromide (EB) reverses pyroptotic markers (reduced LDH release and cleaved caspase-1/GSDMD), confirming a causal role for mtDNA. This step is supported by Dang et al. (2017), who reported that mtDNA–AIM2 interactions drive pyroptosis and identified mtDNA as a key DAMP for AIM2 inflammasome activation.

Multi-level rescue framework. The conclusions above are supported by a convergent, multi-level rescue strategy that targets each node of the self-perpetuating axis. At the sensor level, neuron-targeted AIM2 knockdown — *in vitro* via lentiviral shAIM2 in primary hippocampal neurons (**Figures 5A–H**) and *in vivo* via AAV-PhP.eB shAIM2 stereotaxically delivered to CA3 (**Figure 5I**; AAV coverage of the dorsoventral CA3 extent and neuron-restricted transduction with a preserved contralateral hemisphere are documented in **Supplementary Figure 9**; **Supplementary Figure 10**) — significantly attenuated pyroptotic markers and rescued cognitive function in three independent hippocampus-dependent tasks (Y-maze, NOR and MWM; **Figure 2**). At the effector level, CRISPR-mediated GSDMD-KO, but not Bax-KO, prevented injury-induced loss of ΔΨm and cytosolic mtDNA accumulation (**Figures 8**, **9**), formally separating GSDMD-driven mitochondrial permeabilization from canonical Bax-mediated MOMP. At the ligand level, two orthogonal mtDNA-depletion strategies — ethidium bromide (EB) and 2′,3′-dideoxycytidine (ddC, a selective POLG inhibitor) — both reversed pyroptotic markers after mechanical injury (**Figure 4** and **Supplementary Figure 7**). At the upstream-caspase level, the caspase-8 inhibitor Z-IETD-FMK attenuated early (0.5–3 h) GSDMD-NT generation after mechanical injury, whereas the caspase-1 inhibitor VX-765 did not (**Figure 6**), pharmacologically separating the upstream initiating step from the downstream inflammasome amplification. Finally, delayed administration of VX-765 (at 6 h post-injury, after the caspase-8-driven first wave) selectively suppressed the late-phase second wave of cytosolic mtDNA and the corresponding LDH release (**Figures 7J, K**), thereby providing direct causal evidence for the self-perpetuating amplification step. Together, these interventions provide convergent loss-of-function and rescue evidence at the ligand, sensor, effector and upstream-caspase levels, *in vitro* and *in vivo*, and additionally close the loop pharmacologically through delayed caspase-1 inhibition.

Collectively, our temporal (0.5–6 h onset), mechanistic (GSDMD-KO specificity) and functional (AIM2 knockdown rescue) data directly demonstrate the self-perpetuating amplification loop — including the second, caspase-1-dependent wave of mtDNA release that we have now captured by delayed VX-765 administration (**Figures 7J, K**) — distinguishing it from linear pathways and highlighting its role in TBI-CD.

Mechanical injury triggers initial GSDMD cleavage in neurons via caspase-8, independently of canonical inflammasome caspase-1. Our inhibitor studies show that GSDMD-NT levels rise from 0.5 h post-injury and remain elevated through 3 h; this early rise is markedly suppressed by the caspase-8 inhibitor Z-IETD-FMK, but not by the caspase-1 inhibitor VX-765 (**Figure 6**). Caspase-8-cleaved GSDMD-NT then inserts into the mitochondrial outer membrane, producing early permeabilization, loss of ΔΨm and cytosolic mtDNA release (3–6 h). Released mtDNA activates the neuronal AIM2 inflammasome, leading to ASC recruitment, caspase-1 activation and amplified GSDMD cleavage that sustains the self-perpetuating amplification loop (**Figures 7J, K**). This caspase-8–initiated mechanism is distinct from glial NLRP3 and neuronal NLRP1 pathways and is consistent with reports of caspase-8–dependent GSDMD cleavage and mitochondrial targeting in non-canonical pyroptosis (19, 29, 30).

Our mechanistic data identify mtDNA as a key endogenous ligand for neuronal AIM2 in this context. qPCR showed a significant rise in cytoplasmic mtDNA, but not nuclear DNA, in neurons at 3–6 h after *in vitro* mechanical injury. Depletion of mtDNA with EB reversed mechanical-injury-induced cell death (LDH release). Through JC-1 staining and gene-knockout experiments, we further clarified the unique role of GSDMD in this process: GSDMD knockout — but not Bax knockout — prevented mechanical-injury-induced ΔΨm loss, supporting a direct role for GSDMD-NT in mitochondrial permeabilization and mtDNA release independent of canonical apoptotic pathways. These findings define a feed-forward circuit in which GSDMD acts both as a pyroptosis executioner and as an upstream amplifier of AIM2 activation through mitochondrial permeabilization.

Our findings position AIM2 as a key sensor in neurons responding to acute mechanical injury in TBI. This contrasts with the well-documented role of the NLRP3 inflammasome, which is activated mainly in glial cells in response to other damage- or pathogen-associated molecular patterns (DAMPs/PAMPs) (31, 32). AIM2 has been implicated in other CNS conditions, such as early oedema in stroke models, and in systemic inflammation (33, 34), but our study specifically demonstrates its direct triggering by mtDNA in neurons during the acute mechanical phase of TBI. Our extensive use of AAV-PhP.eB-mediated neuron-targeted AIM2 knockdown reinforces the specificity of this neuron-intrinsic AIM2 contribution. Moreover, our identification of GSDMD-NT as a mediator of mitochondrial dysfunction and mtDNA leakage adds a new dimension to GSDMD biology, extending its role beyond plasma-membrane permeabilization to include intracellular amplification of inflammation and supporting recent evidence in other systems (35, 36).

Based on this pathway, we validated the feasibility of targeted intervention. Selective knockdown of AIM2 in hippocampal CA3 neurons using stereotaxically delivered AAV vectors significantly reduced CCI-induced neuronal loss (increased NeuN-positive cells) and pro-inflammatory cytokine release (IL-1β, IL-18) in this region, and markedly improved cognitive performance in the Y-maze, NOR and MWM tasks at 28 days post-injury. This result supports the neuronal AIM2 inflammasome pathway as a key driver of TBI-related cognitive dysfunction. A targeted, neuron- and region-specific intervention may have advantages over broad-spectrum anti-inflammatory drugs, which risk systemic immunosuppression (35), or antioxidants, which may not effectively interrupt the mtDNA–AIM2 cycle (36), and suggests that precise modulation of neuronal AIM2 activity could be a promising therapeutic strategy for TBI.

Although AAV-PhP.eB exhibits high neuronal tropism, minor off-target transduction of glial cells cannot be excluded (37). In principle, off-target knockdown in microglia or astrocytes could contribute to neuroprotection by reducing the global release of IL-1β and IL-18 and thereby mitigating secondary neurotoxicity. However, microglial or astrocytic AIM2 activation after TBI has not been widely reported. Consistently, we show that AIM2 inflammasome activation in the acute phase of TBI is predominantly localized to neurons (**Figure 3**). Our *in vitro* data in isolated neuronal cultures further confirm that AIM2 knockdown rescues neurons from mechanical-injury-induced pyroptosis in a cell-autonomous manner, independent of glia (**Figure 5**).

Unlike NLRP1, a well-characterized neuronal inflammasome in TBI that is primarily activated by mechanical-injury-induced proteotoxic stress, ATP or K^+^ efflux through pannexin-1 channels and drives early caspase-1/GSDMD-mediated pyroptosis (5, 38), our neuron-specific AIM2 pathway is triggered downstream by GSDMD-NT-mediated mitochondrial permeabilization and cytosolic mtDNA release. This positions AIM2 as a distinct dsDNA sensor that contributes to the self-perpetuating amplification loop in the later phase of neuronal injury, rather than serving as the initial mechanical-stress sensor. Recent studies confirm the predominant neuronal expression of NLRP1 and its contribution to acute neuronal loss after CCI, while also highlighting mtDNA-driven AIM2 activation as an additional, non-redundant mechanism for sustained pyroptosis and cognitive decline (5, 39).

Bioinformatic corroboration across mouse, rat and human TBI transcriptomic datasets. To externally test whether the GSDMD–AIM2 inflammasome axis is engaged at the transcriptional level after TBI, we re-mined five independent, publicly available datasets covering three species and three time windows (**Supplementary Figure 11**). At the early time window (4–6 h), neither mouse hippocampal dentate-gyrus bulk RNA-seq 4 h after fluid percussion injury nor mouse cortical snRNA-seq 6 h after controlled cortical impact (GSE277487, n = 67,191 neurons identified by strict Snap25^+^ marker gating) showed significant induction of Aim2, Gsdmd, Casp8, Casp1 or Nlrp3 — consistent with the view that AIM2 inflammasome activity is regulated post-transcriptionally on a minutes-to-hours timescale rather than at the transcript level. In rat hippocampal bulk RNA-seq 1 day and 14 days after lateral fluid percussion injury, all five detectable inflammasome components — Gsdmd, Casp8, Casp1, Nlrp3 and Nlrp1a — were positively up-regulated in TBI versus Sham at both 1 day (log_2_FC = +0.66, +0.94, +1.98, +1.36, +0.90) and 14 days (log_2_FC = +0.73, +0.76, +0.78, +1.41, +0.49; Casp1 P_adj = 0.050 at 14 d). Most importantly, in human cortical snRNA-seq from severe acute TBI patients with surgical-evacuation tissue collected 4 h to 8 d after injury (Garza et al., Cell Reports 2023; GSE209552; n = 12 TBI + 5 controls; 8,020 strictly gated SNAP25^+^ neurons), CASP8 (log_2_FC = +0.047, P_adj = 4.3 × 10^-^³) and NLRP1 (log_2_FC = +0.31, P_adj = 0.050) were significantly up-regulated, and AIM2 showed the strongest positive log_2_FC of any inflammasome component examined (+0.99), although the formal Wilcoxon adjusted P-value was non-significant owing to the extreme sparsity of AIM2 transcripts in single nuclei. This cross-species, cross-time-window pattern indicates that transcriptional induction of the AIM2 inflammasome axis in TBI cortex/hippocampus emerges between 6 h and 24 h, is sustained out to 14 days, and is recapitulated in human acute TBI brain — precisely overlapping the 24 h window at which we performed our immunofluorescence, biochemistry and AAV-shAIM2 rescue experiments. Together with the post-transcriptional engagement we demonstrate at the protein, biochemical and genetic-rescue levels, these public data argue against a model- or batch-specific artefact and support the cross-species generalizability of the neuron-engaged AIM2 axis described here. A complementary analysis of the largest mouse cortex+hippocampus snRNA-seq dataset to date (GSE226072, Day 1/2/3 post-CCI; n = 143,303 strictly gated Snap25^+^ neurons across 48 samples) likewise showed no significant induction of any inflammasome component (all P_adj ≥ 0.16), indicating that the absence of mRNA signal in mouse sc/sn-seq reflects a species- and model-specific feature of the transcriptional response rather than a technical limitation (insufficient n, sparse detection or power).

Protein-level versus transcript-level regulation of AIM2 in acute TBI. We emphasize that the principal evidence for neuronal AIM2 engagement after TBI in the present study is at the protein level — quantitative immunofluorescence and Western blot of AIM2, ASC speck assembly, cleaved caspase-1 (p20) and GSDMD-NT, together with AAV-mediated AIM2 knockdown that abrogates these protein-level readouts and rescues cognitive function (**Figures 2**, **3**, **5**). The lack of significant acute (4–6 h) AIM2 mRNA induction in mouse cortical and hippocampal scRNA/snRNA-seq datasets (**Supplementary Figure 11C**) therefore does not contradict our findings. It is fully consistent with the established literature in which inflammasome activity — including that of the AIM2 inflammasome — is regulated primarily at the post-translational level (oligomerization around cytosolic dsDNA ligands, ASC speck nucleation and caspase-1 auto-cleavage) rather than purely through transcriptional induction of inflammasome-component genes (9, 32). Pre-existing basal pools of AIM2 protein in neurons can be rapidly engaged by injury-induced cytosolic mtDNA without requiring an immediate mRNA upregulation; sustained inflammasome activity and the broader inflammatory program then drive transcriptional reinforcement at later timepoints (24 h to 14 d), as evidenced by the rat hippocampus and human acute TBI datasets analyzed above. The cross-species transcriptomic analysis therefore complements rather than contradicts our protein- and function-level evidence for an early-acute neuronal AIM2 inflammasome axis.

While immunofluorescence shows that GSDMD is predominantly co-localized with NeuN-positive neurons, partial overlap with astrocytes was also observed (**Supplementary Figure 5**). Recent studies have highlighted astrocytic GSDMD expression in the CNS, where it may amplify secondary inflammation through IL-1β and IL-18 release (40). In infectious CNS diseases, astrocytic GSDMD is primarily activated via the NLRP3 inflammasome, a pathway parallel to the neuronal AIM2 inflammasome. Although TBI may also trigger NLRP3/GSDMD signaling in astrocytes (40), this does not diminish AIM2’s role as the initiator and amplifier of inflammatory signaling in neurons after injury. We therefore propose that the observed cognitive recovery is primarily driven by direct preservation of neuronal viability through blockade of the neuron-intrinsic GSDMD–mtDNA–AIM2 loop, with reduced glial inflammation serving as a secondary, supportive mechanism.

Our study identifies the AIM2 inflammasome in neurons as a potential driver of neuronal injury, but we acknowledge the possible involvement of other DNA-sensing machineries in TBI pathology. The release of mtDNA is a ‘promiscuous’ signal that could engage cGAS–STING or TLR9. Indeed, cGAS–STING activation has been reported to promote neuroinflammation through the IFN-β axis in TBI models (28), and NLRP3 is a canonical sensor of cellular stress often activated in microglia (31). Several lines of evidence in our study argue, however, for AIM2 dominance in mediating acute neuronal pyroptosis. First, TLR9 is predominantly endosomal and typically senses extracellular DNA taken up by phagocytes (e.g. microglia), whereas our data show direct mtDNA leakage into the neuronal cytosol — the compartment surveyed by AIM2. Second, cGAS–STING activation depends on a transcriptional program producing interferons, which is temporally distinct from the rapid GSDMD cleavage and LDH release (pyroptosis) we observe within hours of mechanical injury. Third, and most importantly, knockdown of AIM2 alone was sufficient to abrogate GSDMD cleavage and rescue cognitive function. If NLRP3 or cGAS pathways were the primary drivers of neuronal death in this context, AIM2 silencing would have yielded only a partial rescue. Other sensors may modulate the inflammatory tone, but the mtDNA–AIM2 axis appears to be the indispensable executioner of acute neuronal loss in this setting.

Acute AIM2-driven neuronal pyroptosis in the hippocampal CA3 region (3–24 h) initiates a multi-level cascade that culminates in chronic network dysfunction and memory impairment at 7–28 days. Cellularly, GSDMD-NT-mediated mtDNA release activates the AIM2 inflammasome and caspase-1, producing pyroptotic death and irreversible CA3 neuronal loss. This disrupts the hippocampal trisynaptic pathway and impairs spatial encoding. Synaptically, DAMP and IL-1β/IL-18 release drives microglial activation, synaptic pruning, reduced long-term potentiation (LTP) and enhanced long-term depression. Inflammation evolves into chronic gliosis. Although neuron-specific AAV-shAIM2 substantially suppresses AIM2 in most CA3 neurons, knockdown is incomplete and some cells remain vulnerable to delayed damage. Full restoration of hippocampus-dependent cognition requires time for adult hippocampal neurogenesis and synaptic integration (typically 4–8 weeks), as well as resolution of parallel injury mechanisms such as Ca²^+^ overload, oxidative stress and excitotoxicity. Pre-injury AIM2 knockdown rescues both acute pyroptotic markers and chronic behavioral deficits (Y-maze, NOR, MWM), but network recovery is gradual. This acute-to-chronic progression is consistent with recent studies linking early inflammasome-driven neuronal loss to sustained circuit remodeling and cognitive impairment (Kim et al., 2020; Poh et al., 2021; de Macedo Filho et al., 2024).

Although our study highlights the role of the mtDNA–AIM2–GSDMD axis in post-TBI cognitive decline, several limitations warrant future investigation. First, the precise route of mtDNA escape from mitochondria remains to be fully elucidated — whether the GSDMD pore is the sole or primary conduit, or whether it cooperates with other mitochondrial channels such as the mPTP or VDAC. Second, although our neuron-specific AAV-AIM2 shRNA strategy showed clear efficacy and specificity within the 28-day study period, its long-term safety and potential off-target effects remain to be assessed. Third, our experiments were performed exclusively in male C57BL/6 mice; sex-dependent differences in inflammasome signaling cannot be excluded and warrant systematic investigation in future work.

A remaining technical limitation concerns the cellular resolution of cytosolic mtDNA quantification: our qPCR-based fractionation approach reports the population-average ratio across cultured neurons rather than single-cell mtDNA dynamics. Live single-cell mtDNA reporters in primary neurons after mechanical injury — though technically demanding — would further refine the temporal model of the self-perpetuating loop.

A technical limitation concerns our AAV-PhP.eB delivery strategy. Although systemic intravenous (i.v.) administration could achieve broader CNS transduction to better match the spatial extent of CCI pathology, it was not feasible here because shRNA expression typically requires 2–4 weeks for maximal knockdown — a window that exceeds the acute AIM2 inflammasome activation phase (mainly within 48 h post-injury, peaking at ~24 h). Post-injury i.v. injection is therefore not practical for timely intervention in this rapid-onset pathway. For future translational efforts, nanoparticle-based platforms hold considerable promise for acute, targeted delivery of AIM2 shRNA or siRNA across the disrupted blood–brain barrier, enabling effective suppression within hours of injury (1–3). Recent advances have demonstrated successful nanoparticle-mediated nucleic acid delivery for inflammasome inhibition in CNS trauma and ischaemia models, offering enhanced BBB penetration, reduced off-target effects and rapid onset suitable for acute TBI therapy (41–43). These approaches represent important next steps for this line of research.

## Conclusions

In summary, this study identifies a neuron-enriched, self-perpetuating GSDMD–mtDNA–AIM2 inflammasome–pyroptosis axis as a driver of cognitive impairment after TBI. Mechanical injury activates this pathway through caspase-8-mediated cleavage of GSDMD into GSDMD-NT, which rapidly triggers mitochondrial permeabilization and mtDNA release within neurons. The released mtDNA then activates the AIM2 inflammasome, generating additional caspase-1-dependent GSDMD cleavage that drives a second, self-perpetuating wave of cytosolic mtDNA — directly demonstrated here by delayed caspase-1 inhibition (**Figures 7J, K**). Targeted inhibition of AIM2 in hippocampal neurons via AAV-mediated knockdown disrupts this pathway, rescues neuronal survival and significantly improves cognitive function. These findings advance our understanding of TBI pathophysiology by defining a cell-autonomous inflammatory cascade that is initiated by caspase-8-driven GSDMD activation and sustained through the self-perpetuating GSDMD–mtDNA–AIM2 axis, positioning AIM2 as a candidate therapeutic target. The work provides a rationale for developing neuroprotective strategies that selectively modulate neuronal inflammasome activity to limit secondary injury and improve long-term outcomes after TBI.

## Data Availability

The original contributions presented in the study are included in the article/**Supplementary Material**. Further inquiries can be directed to the corresponding author.

## Supplementary material

Supplementary Figure 1 Chronic neuroinflammation in the hippocampus after CCI. Representative immunofluorescence images and quantification of microglial (Iba1) and astrocytic (GFAP) reactivity in the ipsilateral hippocampal CA3 region of Sham and CCI mice at 7, 14 and 28 days post-injury. **(A)** Representative confocal images; the CA3 sub-region is outlined by a white dotted line; nuclei are counterstained with DAPI. Scale bar, 50 μm. **(B)** Quantification of Iba1-positive area fraction. **(C)** Quantification of GFAP-positive area fraction. Data are mean ± SEM; n = 3–4 mice per group per time point. *P < 0.05, **P < 0.01 vs. Sham. Statistical analysis: two-way ANOVA with Šídák’s *post hoc* test.

Supplementary Figure 2 Validation of AAV-PhP.eB-mediated AIM2 knockdown in hippocampal CA3 neurons. Mice received stereotaxic injection of AAV-PhP.eB-shAIM2 (carrying an RFP reporter under the U6 promoter) or AAV-PhP.eB-shScr into the ipsilateral hippocampal CA3 region two weeks before CCI. **(A–C)** Representative confocal images of the CA3 region stained for RFP (AAV reporter, red), the neuronal marker NeuN (green) and DAPI (blue). The CA3 sub-region is outlined by a white dotted line. Scale bar, 50 μm (main panels); 20 μm (insets). **(D)** Quantification of RFP-positive cells co-expressing NeuN, GFAP or Iba1, demonstrating predominantly neuronal transduction (RFP/NeuN > 90%; RFP/GFAP < 5%; RFP/Iba1 < 5%). Data are mean ± SEM; n = 3 mice/group. Statistical analysis: one-way ANOVA with Tukey’s *post hoc* test.

Supplementary Figure 3 Mechanical injury triggers AIM2 inflammasome activation in differentiated Neuro-2a (N2a) cells. Differentiated N2a cells were subjected to *in vitro* mechanical scratch injury and harvested at 3, 6, 12 and 24 h. **(A)** Representative Western blots of AIM2, cleaved caspase-1 (p20), GSDMD and GSDMD-NT; β-actin served as the loading control. **(B–D)** Quantification of corresponding protein levels, normalised to the control (Con) group. **(E)** LDH release into the supernatant at the indicated time points. Data are mean ± SEM from three independent experiments. *P < 0.05, **P < 0.01, ***P < 0.001 vs. Con. Statistical analysis: one-way ANOVA with Tukey’s *post hoc* test.

Supplementary Figure 4 CRISPR/Cas9-mediated knockout of Bax and Gsdmd in primary hippocampal neurons. Primary hippocampal neurons (DIV 4–5) were transduced with lentiviral SpCas9 plus single-guide RNAs targeting Bax or Gsdmd, or with a non-targeting sgRNA control. No antibiotic selection was applied. **(A)** Western blot validation of Bax-KO and GSDMD-KO at the protein level (>70% reduction), with β-actin as the loading control. **(B)** Quantification of Bax and GSDMD protein levels relative to the non-targeting control. **(C)** Cell viability of transduced neurons by MTT assay (>90% viability across n = 3–5 cultures). **(D)** Representative Sanger chromatograms across the sgRNA-targeted loci confirming on-target indels. Data are mean ± SEM from three independent cultures. ***P < 0.001 vs. non-targeting sgRNA. Statistical analysis: one-way ANOVA with Tukey’s *post hoc* test.

Supplementary Figure 5 GSDMD shows predominant neuronal localisation with detectable astrocytic and minimal microglial overlap in CA3 after CCI. **(A)** Representative confocal images of GSDMD (green) colocalising with the neuronal marker NeuN (red) in the CA3 region of Sham and CCI mice 24 h post-injury. Nuclei counterstained with DAPI (blue). Scale bar, 50 μm. **(B)** Representative confocal images of GSDMD (green) colocalising with the astrocyte marker GFAP (red) in the same region; scale bar, 50 μm. **(C)** Representative confocal images of GSDMD (green) colocalising with the microglia marker Iba1 (red) in the same region; scale bar, 50 μm. Quantification (right of each row) shows the fraction of GSDMD-positive cells that overlap with NeuN, GFAP or Iba1 (n = 3 images per group from 3 mice). Data are mean ± SEM. **P < 0.01, ***P < 0.001 vs. Sham. Statistical analysis: unpaired two-tailed Student’s t-test for each cell-type marker.

Supplementary Figure 6 GSDMD subcellular localisation dynamics after mechanical injury: mitochondrial targeting precedes plasma-membrane insertion. Primary hippocampal neurons (DIV 7–10) were subjected to *in vitro* mechanical injury and fixed at 3 h or 12 h post-injury. **(A)** Representative confocal images showing GSDMD (orange) co-localisation with the mitochondrial outer-membrane marker TOM70 (green) at 3 h and 12 h post-injury compared with the uninjured control. Scale bar, 10 μm. Note the early increase in GSDMD–TOM70 co-localisation at 3 h, which remains apparent and trends upward at 12 h. **(B)** Representative confocal images showing GSDMD (orange) co-localisation with the plasma-membrane marker α1-Na^+^/K^+^-ATPase (green) at 3 h and 12 h post-injury compared with the uninjured control. Scale bar, 10 μm. Note the lack of apparent co-localisation at 3 h, with marked enhancement at 12 h. These findings support the temporal dynamics of GSDMD translocation, prioritising mitochondrial targeting early after injury before plasma-membrane insertion, consistent with the proposed GSDMD–mtDNA–AIM2 inflammasome amplification loop in neuronal pyroptosis.

Supplementary Figure 7 Orthogonal pharmacological mtDNA depletion with ddC attenuates injury-induced LDH release. Primary hippocampal neurons (DIV 7–10) were pre-treated with the POLG-selective inhibitor 2′,3′-dideoxycytidine (ddC, 10 μM, 5-day pre-treatment; ~70–80% mtDNA knock-down confirmed by qPCR) and then subjected to *in vitro* mechanical injury. Relative LDH release was measured at 6 h post-injury in four groups: untreated control (NC), NC + mechanical injury (NC-Trauma), ddC alone and ddC + mechanical injury (ddC-Trauma). ddC pre-treatment significantly suppressed injury-induced LDH release (*P < 0.05 versus NC-Trauma), recapitulating the ethidium bromide phenotype shown in main **Figure 7C**–I through a mechanistically distinct enzymatic route and excluding off-target effects of EB as an explanation. Data are mean ± SEM from n ≥ 5 independent biological replicates; one-way ANOVA with Tukey’s *post-hoc* test. EB and ddC therefore constitute two orthogonal pharmacological approaches that converge on the same mtDNA-dependent activation mechanism of the neuronal AIM2 inflammasome.

Supplementary Figure 8 Baseline neurological, motor and visual function do not differ between groups prior to behavioural testing. One week before CCI, all mice underwent baseline assessments to rule out confounding pre-existing impairments that could affect the cognitive measurements in **Figure 2** | **(A)** Neurological severity score (NSS) at baseline (mean NSS ≈ 0 across all groups, indicating minimal motor deficits). **(B)** Escape latency to the platform in the Morris water maze (MWM). **(C)** Average swim speed during MWM acquisition. Data are mean ± SEM; n = 6–10 mice/group. n.s., not significant by one-way ANOVA. These data confirm the absence of pre-existing motor, cognitive or visual deficits that could confound the post-injury cognitive measurements.

Supplementary Figure 9 AAV-PhP.eB-mediated transduction efficiently covers the dorsoventral extent of the hippocampal CA3 sub-region. Continuous coronal sections (30 μm, every 5th section) through the ipsilateral hippocampus, two weeks after stereotaxic injection of AAV-PhP.eB carrying an RFP reporter under the U6 promoter (titer ≈ 1 × 10¹³ vg/mL). Representative low-magnification confocal mosaic showing RFP signal (red) and DAPI (blue) across the rostrocaudal extent of CA3. Note continuous RFP coverage along the dorsoventral axis, confirming that the single stereotaxic injection produces anatomically broad transduction across the CA3 sub-region targeted by our behavioural and biochemical analyses (**Figure 5** and **2**). Scale bar, 200 μm.

Supplementary Figure 10 Triple immunolabelling confirms neuron-restricted AIM2 knockdown and a preserved contralateral hemisphere after stereotaxic AAV-PhP.eB-shAIM2 injection. Mice received unilateral stereotaxic injection of AAV-PhP.eB-shAIM2 (RFP reporter; titer ≈ 1 × 10¹³ vg/mL) or AAV-PhP.eB-shScramble into the ipsilateral CA3 two weeks before CCI. Brains were collected 28 days post-CCI. **(A)** Representative confocal images of the ipsilateral CA3 region triple-labelled for the AAV reporter RFP (red), AIM2 (green) and the neuronal marker NeuN (blue). RFP co-localises predominantly with NeuN (>95% co-localisation in cell-type quantification), while in shAIM2-transduced (RFP-positive) neurons AIM2 immunoreactivity is markedly reduced or absent, demonstrating successful neuron-restricted knockdown. Scale bar, 50 μm (main panels); 20 μm (insets). **(B)** Representative confocal images of the contralateral hippocampal CA3 region from the same animals, showing no detectable AIM2 upregulation and preserved NeuN-positive neuronal density, supporting that the observed therapeutic effects are locally driven by the focal ipsilateral CCI and the unilateral AAV intervention. Scale bar, 50 μm. **(C)** Additional co-staining of RFP with the microglial marker Iba1 and the astrocytic marker GFAP (separate sections from the same brains) confirms negligible AAV transduction of microglia or astrocytes (<5% overlap), establishing the neuron-restricted tropism of the AAV-PhP.eB delivery used throughout this study. Scale bar, 50 μm.

Supplementary Figure 11 Transcriptional kinetics of the GSDMD–AIM2 inflammasome axis across mouse, rat and human TBI scRNA/snRNA and bulk RNA-seq datasets. Five publicly available datasets covering three species (mouse, rat, human) and three time windows (4–6 h, 24 h–14 d, acute human 4 h–8 d) were re-mined to externally test whether key components of the proposed GSDMD–AIM2 axis are induced at the mRNA level after TBI. **(A)** Schematic of the transcriptional kinetics emerging from this cross-dataset analysis: at 4–6 h post-injury the mRNA response is statistically silent; between 24 h and 14 days all five detectable inflammasome components (Gsdmd, Casp8, Casp1, Nlrp3, Nlrp1a) are positively up-regulated in rat hippocampus; and in human acute TBI brain CASP8/NLRP1 are significantly up-regulated while AIM2 shows the strongest positive log_2_FC. The arrow marks the 24 h window at which our immunofluorescence, Western blot and AAV-shAIM2 rescue experiments were performed. **(B)** Catta-Preta et al. (44) (GSE173975): rat hippocampal bulk RNA-seq after lateral fluid percussion injury at 1 day (TBI n = 4 vs Sham n = 2) and 14 days (TBI n = 4 vs Sham n = 4); † marks 0.05 < P_adj < 0.10 (Welch’s t on log_2_(CPM + 1) with Benjamini–Hochberg FDR across 17,170 detected genes). Aim2 and Nlrp1b were below the rat-bulk detection floor. **(C)** Early-window mouse datasets in which mRNA response is not yet engaged: Denninger et al. (45) (GSE189574), mouse DG bulk RNA-seq 4 h after lateral fluid percussion injury (TBI n = 3 vs Sham n = 3); and GSE277487, mouse cortical snRNA-seq 6 h after controlled cortical impact (TBI n = 3 vs Ctrl n = 3), neurons identified by strict single-cell marker gating (SNAP25^+^ ∩ {SLC17A7^+^ ∪ GAD1^+^ ∪ CAMK2A^+^}; n = 67,191 strict neurons; TBI: Ctrl ratio 0.85:1); all P_adj > 0.99. **(D)** Garza et al. (46) (GSE209552): human cortical snRNA-seq from severe-acute-TBI patients with surgical-evacuation tissue (n = 12 TBI patients + 5 post-mortem controls; tissue collected 4 h to 8 d after injury). Bar chart of log_2_(fold change) TBI vs Control for the six inflammasome components detectable in human FPKM-filtered nuclei (excluding Nlrp1a/Nlrp1b which lack 1:1 orthologs); CASP8 P_adj = 4.3 × 10^-^³ (*), NLRP1 P_adj = 0.050 (†). AIM2 showed the strongest positive log_2_FC (+0.99) but the formal Wilcoxon adjusted P-value was non-significant due to extreme sparsity of AIM2 transcripts across the 8,020 strictly gated SNAP25^+^ neurons. **(E)** Cross-species, cross-time-window summary heatmap of log_2_(fold change) across the five datasets. Numerical labels show the log_2_FC value with significance code (* P_adj < 0.05; † P_adj < 0.10). NA indicates genes below the detection floor or lacking a 1:1 ortholog. Statistical analysis: Welch’s t-test on log-transformed normalised counts for bulk datasets; Wilcoxon rank-sum test for sc/snRNA-seq datasets; all with Benjamini–Hochberg FDR correction. The complete per-dataset differential-expression tables and the analysis scripts are deposited as Source Data S11.

Supplementary Figure 12 Quantitative colocalization coefficients for **Figure 3A** (AIM2/NeuN) and **Figure 3G** (GSDMD/NeuN). Bar charts showing Pearson’s correlation coefficient (r) and Mander’s overlap coefficients (M1, M2), computed for the representative confocal images presented in main **Figures 3A and G** using a Python implementation of Costes’ regression-based automatic thresholding, validated against the Fiji Coloc2 plugin. **(A)** AIM2/NeuN coefficients across Cortex and CA3 (Sham vs CCI). **(B)** GSDMD/NeuN coefficients across Cortex and CA3 (Sham vs CCI). M1 = fraction of total AIM2/GSDMD signal located within NeuN-positive pixels (≈ 1.0 in CCI panels indicates near-complete neuronal localization). M2 = fraction of total NeuN signal that is co-positive for AIM2/GSDMD. Raw.czi files (n = 8), per-panel coefficient table, and the reproducible Python script are deposited as **Figure 3** source data.
