# Supplementary figures and images for: A self-perpetuating neuron-intrinsic GSDMD–mtDNA–AIM2 inflammasome axis drives neuronal pyroptosis and cognitive impairment after traumatic brain injury
Source: Front Immunol. 2026 Jun 19;17:1867920. doi: 10.3389/fimmu.2026.1867920 (PMC13327905; doi:10.3389/fimmu.2026.1867920)

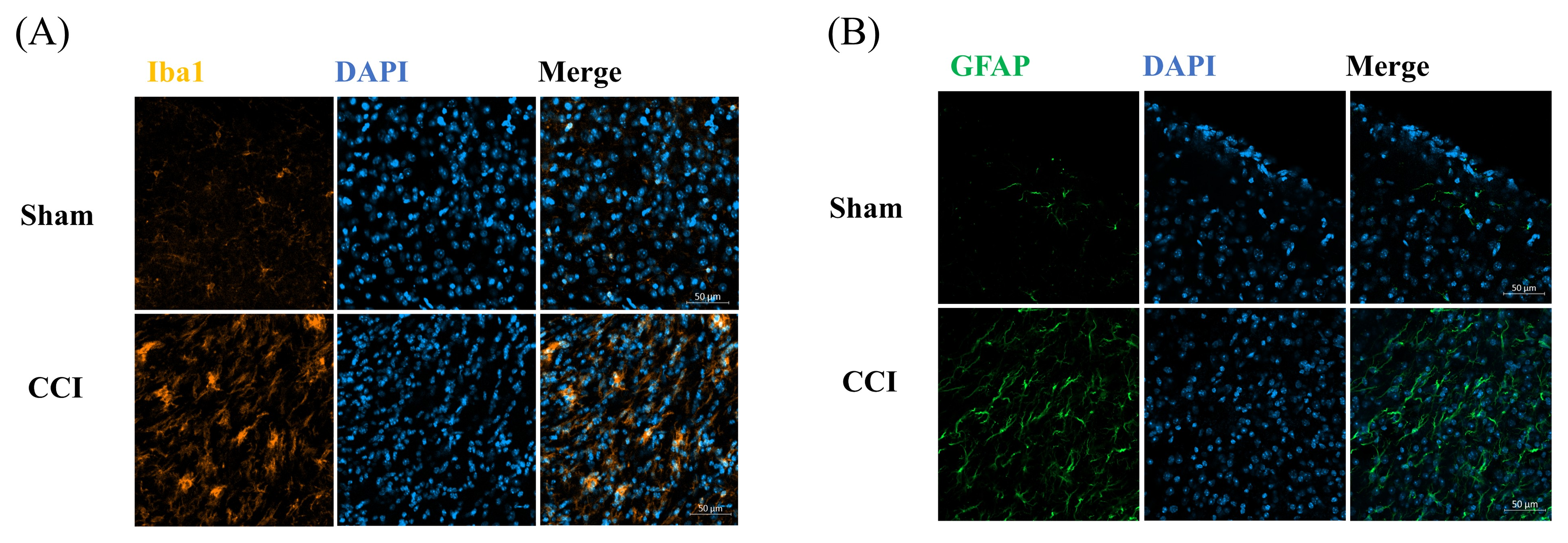

Supplement: Supplementary Figure 1 — Chronic neuroinflammation in the hippocampus after CCI. Representative immunofluorescence images and quantification of microglial (Iba1) and astrocytic (GFAP) reactivity in the ipsilateral hippocampal CA3 region of Sham and CCI mice at 7, 14 and 28 days post-injury. (A) Representative confocal images; the CA3 sub-region is outlined by a white dotted line; nuclei are counterstained with DAPI. Scale bar, 50 μm. (B) Quantification of Iba1-positive area fraction. (C) Quantification of GFAP-positive area fraction. Data are mean ± SEM; n = 3–4 mice per group per time point. *P < 0.05, **P < 0.01 vs. Sham. Statistical analysis: two-way ANOVA with Šídák’s post hoc test. [file Image1.png]

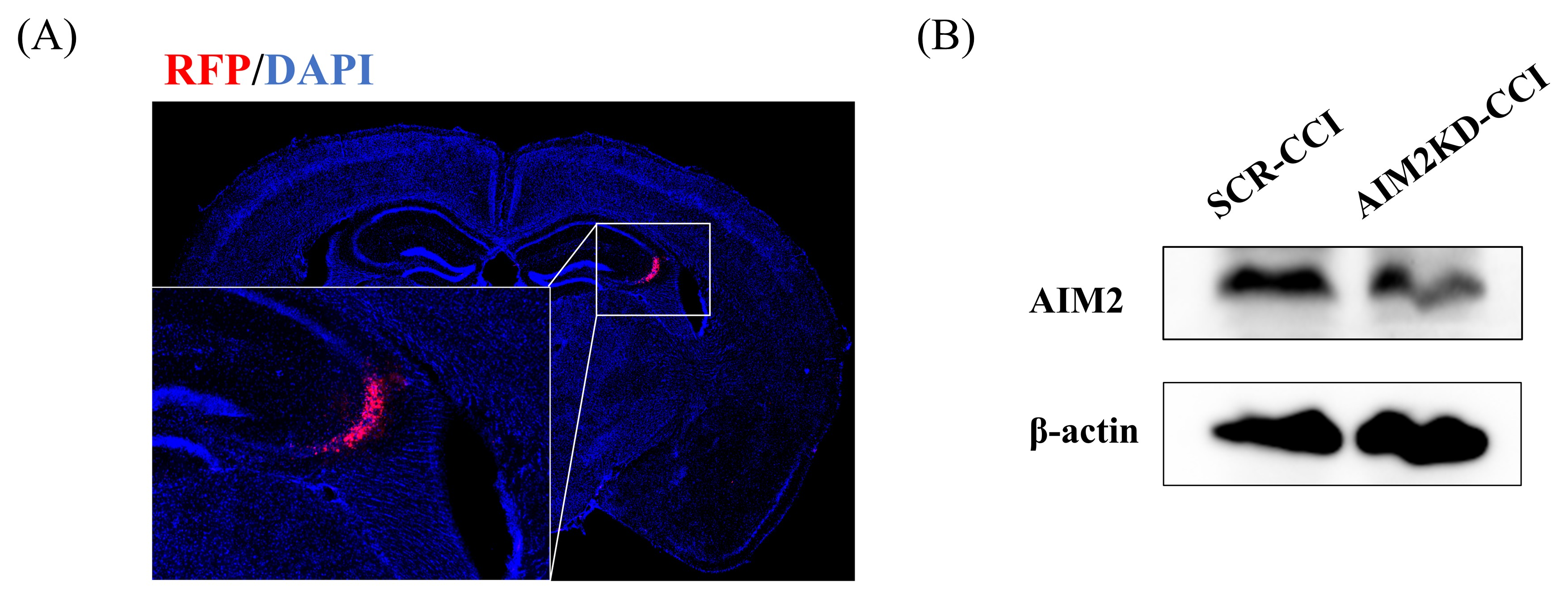

Supplement: Supplementary Figure 2 — Validation of AAV-PhP.eB-mediated AIM2 knockdown in hippocampal CA3 neurons. Mice received stereotaxic injection of AAV-PhP.eB-shAIM2 (carrying an RFP reporter under the U6 promoter) or AAV-PhP.eB-shScr into the ipsilateral hippocampal CA3 region two weeks before CCI. (A–C) Representative confocal images of the CA3 region stained for RFP (AAV reporter, red), the neuronal marker NeuN (green) and DAPI (blue). The CA3 sub-region is outlined by a white dotted line. Scale bar, 50 μm (main panels); 20 μm (insets). (D) Quantification of RFP-positive cells co-expressing NeuN, GFAP or Iba1, demonstrating predominantly neuronal transduction (RFP/NeuN > 90%; RFP/GFAP < 5%; RFP/Iba1 < 5%). Data are mean ± SEM; n = 3 mice/group. Statistical analysis: one-way ANOVA with Tukey’s post hoc test. [file Image2.png]

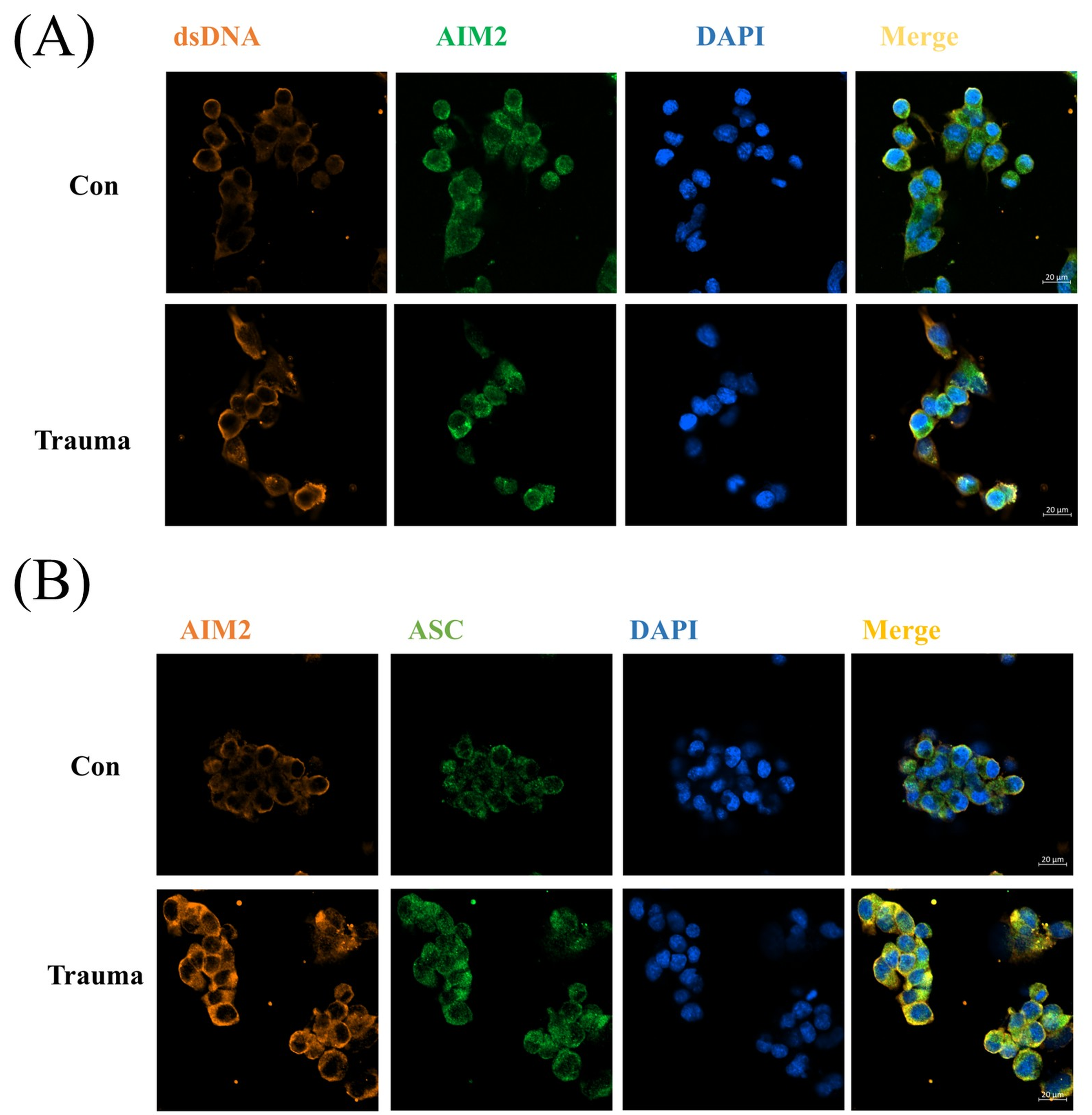

Supplement: Supplementary Figure 3 — Mechanical injury triggers AIM2 inflammasome activation in differentiated Neuro-2a (N2a) cells. Differentiated N2a cells were subjected to in vitro mechanical scratch injury and harvested at 3, 6, 12 and 24 h. (A) Representative Western blots of AIM2, cleaved caspase-1 (p20), GSDMD and GSDMD-NT; β-actin served as the loading control. (B–D) Quantification of corresponding protein levels, normalised to the control (Con) group. (E) LDH release into the supernatant at the indicated time points. Data are mean ± SEM from three independent experiments. *P < 0.05, **P < 0.01, ***P < 0.001 vs. Con. Statistical analysis: one-way ANOVA with Tukey’s post hoc test. [file Image3.png]

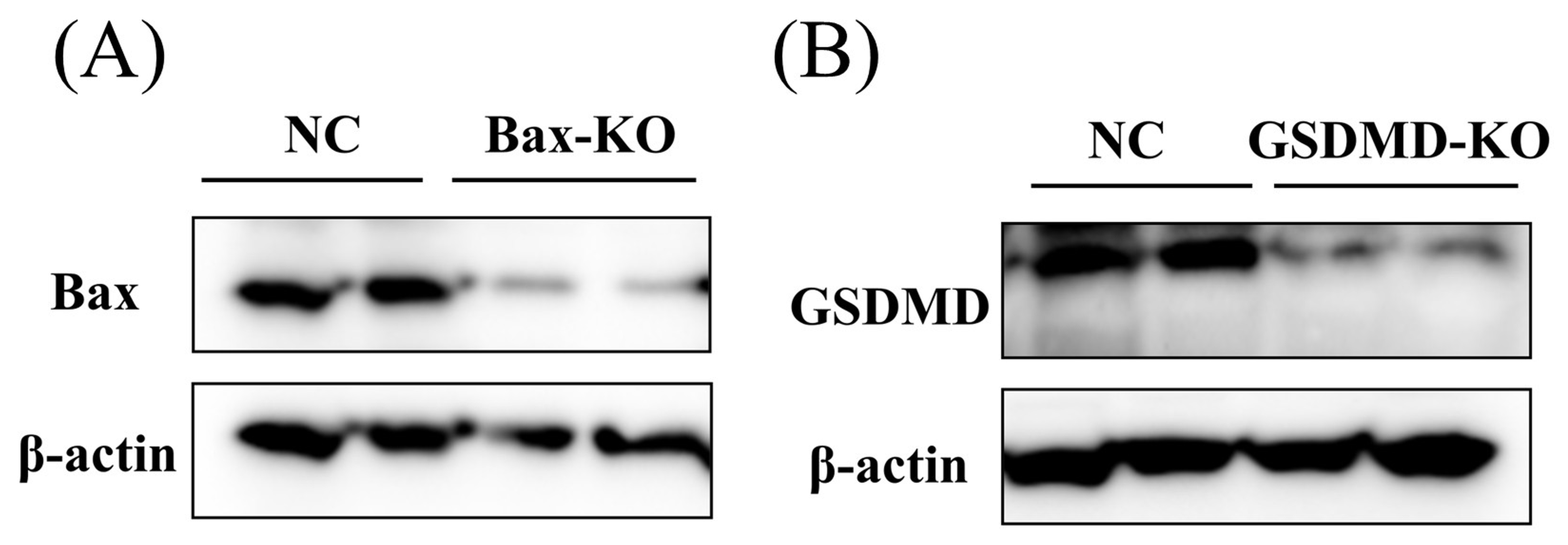

Supplement: Supplementary Figure 4 — CRISPR/Cas9-mediated knockout of Bax and Gsdmd in primary hippocampal neurons. Primary hippocampal neurons (DIV 4–5) were transduced with lentiviral SpCas9 plus single-guide RNAs targeting Bax or Gsdmd, or with a non-targeting sgRNA control. No antibiotic selection was applied. (A) Western blot validation of Bax-KO and GSDMD-KO at the protein level (>70% reduction), with β-actin as the loading control. (B) Quantification of Bax and GSDMD protein levels relative to the non-targeting control. (C) Cell viability of transduced neurons by MTT assay (>90% viability across n = 3–5 cultures). (D) Representative Sanger chromatograms across the sgRNA-targeted loci confirming on-target indels. Data are mean ± SEM from three independent cultures. ***P < 0.001 vs. non-targeting sgRNA. Statistical analysis: one-way ANOVA with Tukey’s post hoc test. [file Image4.png]

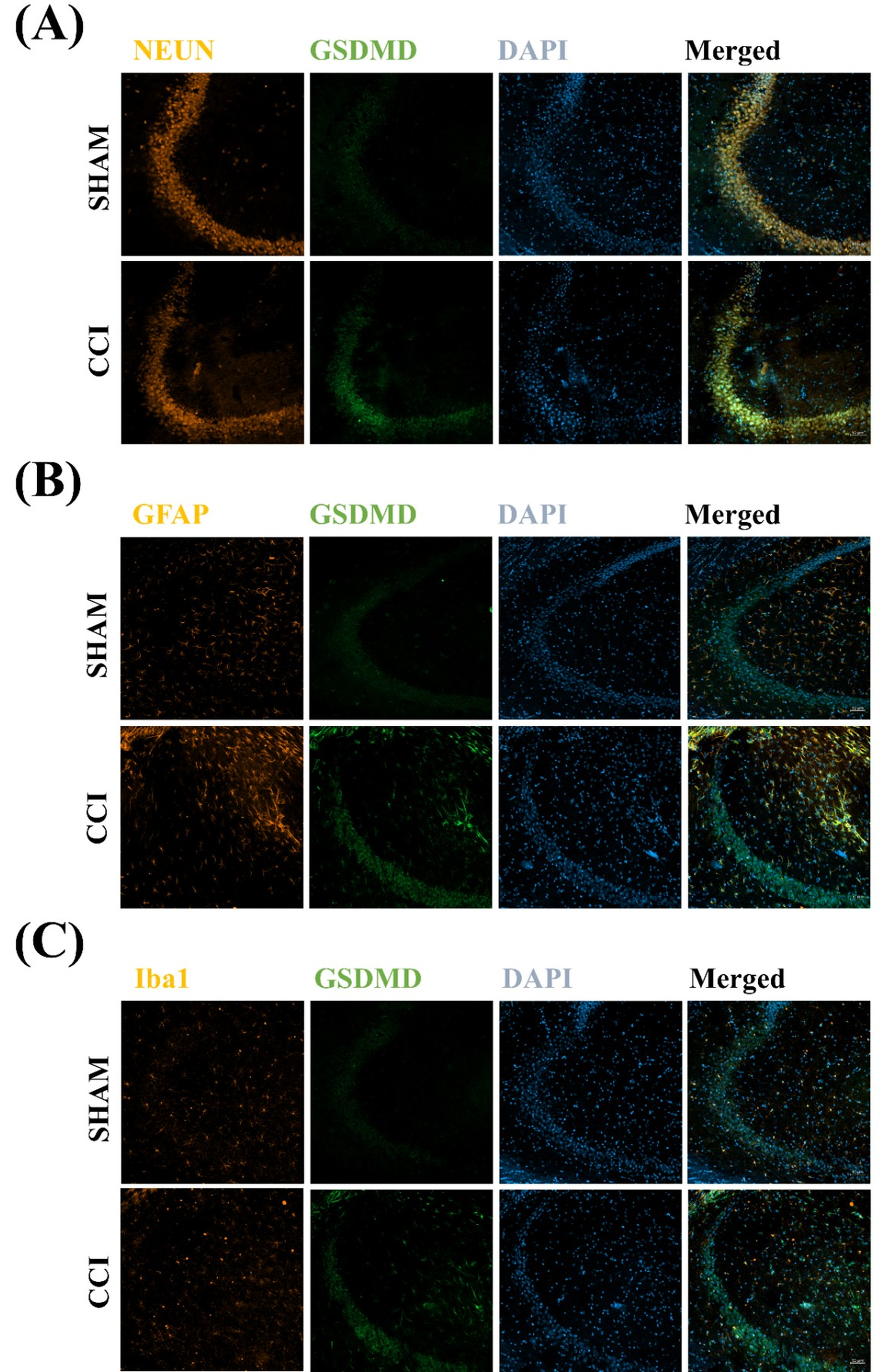

Supplement: Supplementary Figure 5 — GSDMD shows predominant neuronal localisation with detectable astrocytic and minimal microglial overlap in CA3 after CCI. (A) Representative confocal images of GSDMD (green) colocalising with the neuronal marker NeuN (red) in the CA3 region of Sham and CCI mice 24 h post-injury. Nuclei counterstained with DAPI (blue). Scale bar, 50 μm. (B) Representative confocal images of GSDMD (green) colocalising with the astrocyte marker GFAP (red) in the same region; scale bar, 50 μm. (C) Representative confocal images of GSDMD (green) colocalising with the microglia marker Iba1 (red) in the same region; scale bar, 50 μm. Quantification (right of each row) shows the fraction of GSDMD-positive cells that overlap with NeuN, GFAP or Iba1 (n = 3 images per group from 3 mice). Data are mean ± SEM. **P < 0.01, ***P < 0.001 vs. Sham. Statistical analysis: unpaired two-tailed Student’s t-test for each cell-type marker. [file Image5.png]

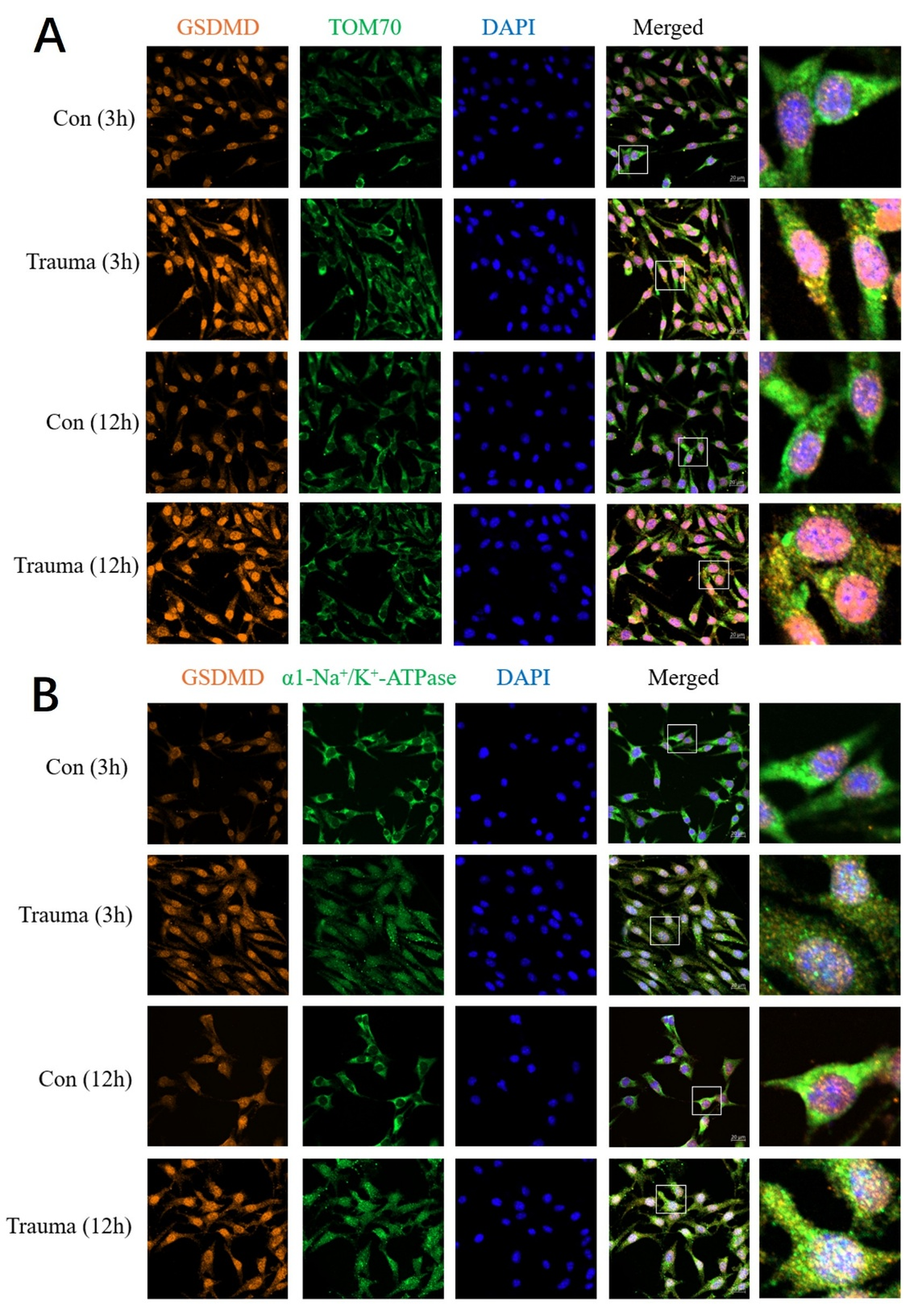

Supplement: Supplementary Figure 6 — GSDMD subcellular localisation dynamics after mechanical injury: mitochondrial targeting precedes plasma-membrane insertion. Primary hippocampal neurons (DIV 7–10) were subjected to in vitro mechanical injury and fixed at 3 h or 12 h post-injury. (A) Representative confocal images showing GSDMD (orange) co-localisation with the mitochondrial outer-membrane marker TOM70 (green) at 3 h and 12 h post-injury compared with the uninjured control. Scale bar, 10 μm. Note the early increase in GSDMD–TOM70 co-localisation at 3 h, which remains apparent and trends upward at 12 h. (B) Representative confocal images showing GSDMD (orange) co-localisation with the plasma-membrane marker α1-Na+/K+-ATPase (green) at 3 h and 12 h post-injury compared with the uninjured control. Scale bar, 10 μm. Note the lack of apparent co-localisation at 3 h, with marked enhancement at 12 h. These findings support the temporal dynamics of GSDMD translocation, prioritising mitochondrial targeting early after injury before plasma-membrane insertion, consistent with the proposed GSDMD–mtDNA–AIM2 inflammasome amplification loop in neuronal pyroptosis. [file Image6.png]

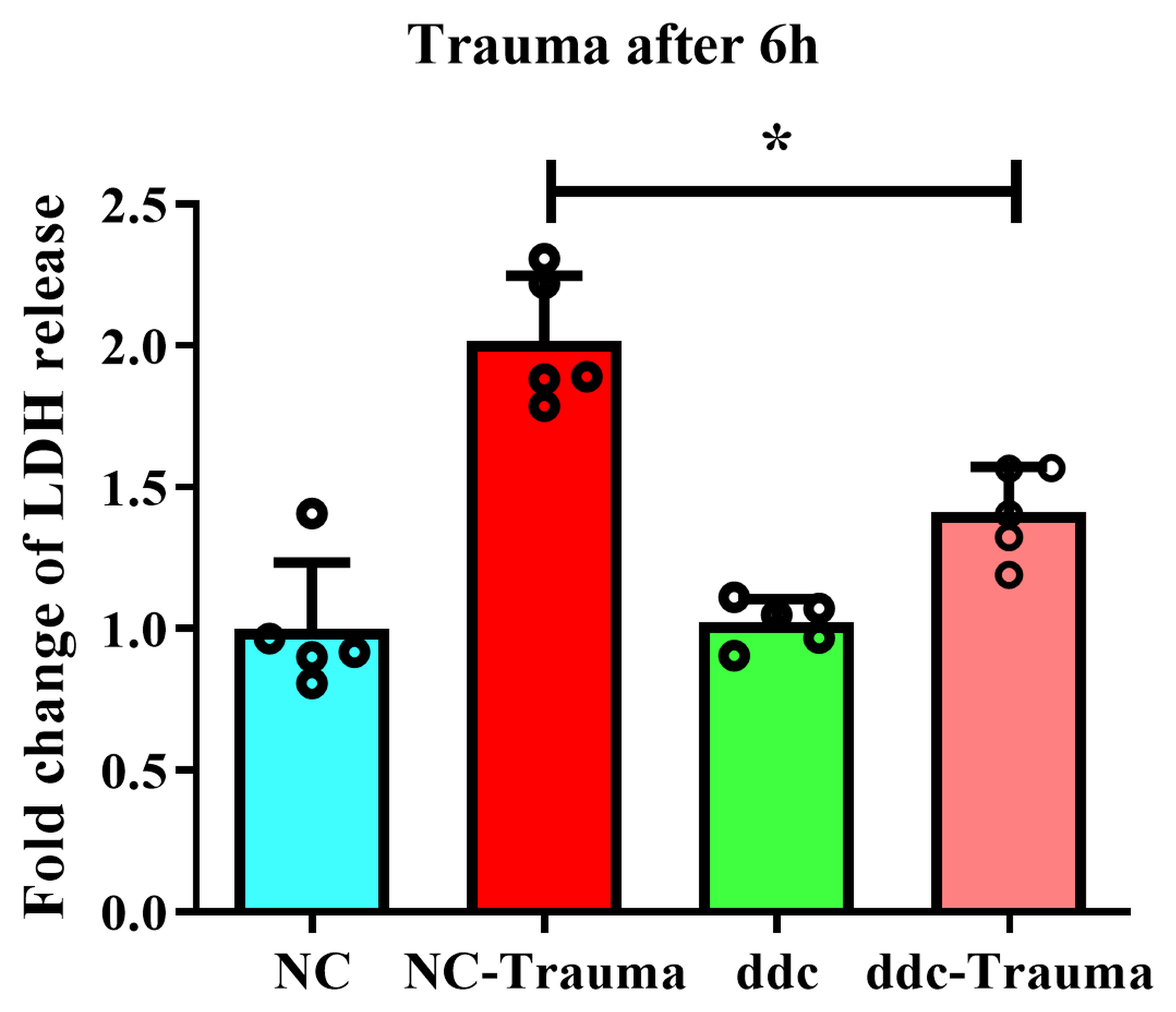

Supplement: Supplementary Figure 7 — Orthogonal pharmacological mtDNA depletion with ddC attenuates injury-induced LDH release. Primary hippocampal neurons (DIV 7–10) were pre-treated with the POLG-selective inhibitor 2′,3′-dideoxycytidine (ddC, 10 μM, 5-day pre-treatment; ~70–80% mtDNA knock-down confirmed by qPCR) and then subjected to in vitro mechanical injury. Relative LDH release was measured at 6 h post-injury in four groups: untreated control (NC), NC + mechanical injury (NC-Trauma), ddC alone and ddC + mechanical injury (ddC-Trauma). ddC pre-treatment significantly suppressed injury-induced LDH release (*P < 0.05 versus NC-Trauma), recapitulating the ethidium bromide phenotype shown in main Figure 7C–I through a mechanistically distinct enzymatic route and excluding off-target effects of EB as an explanation. Data are mean ± SEM from n ≥ 5 independent biological replicates; one-way ANOVA with Tukey’s post-hoc test. EB and ddC therefore constitute two orthogonal pharmacological approaches that converge on the same mtDNA-dependent activation mechanism of the neuronal AIM2 inflammasome. [file Image7.png]

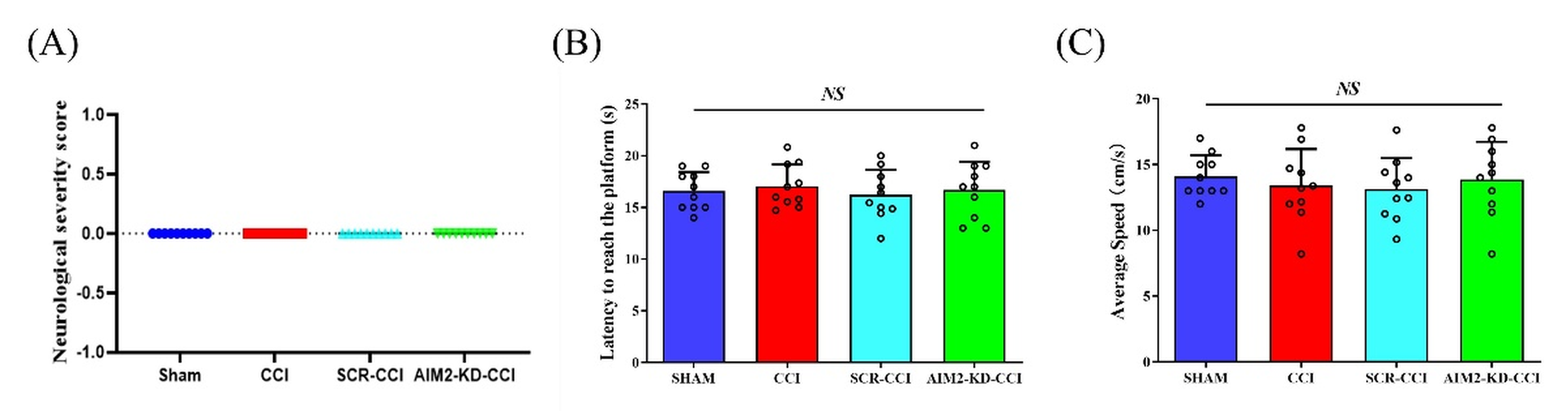

Supplement: Supplementary Figure 8 — Baseline neurological, motor and visual function do not differ between groups prior to behavioural testing. One week before CCI, all mice underwent baseline assessments to rule out confounding pre-existing impairments that could affect the cognitive measurements in Figure 2 | (A) Neurological severity score (NSS) at baseline (mean NSS ≈ 0 across all groups, indicating minimal motor deficits). (B) Escape latency to the platform in the Morris water maze (MWM). (C) Average swim speed during MWM acquisition. Data are mean ± SEM; n = 6–10 mice/group. n.s., not significant by one-way ANOVA. These data confirm the absence of pre-existing motor, cognitive or visual deficits that could confound the post-injury cognitive measurements. [file Image8.png]

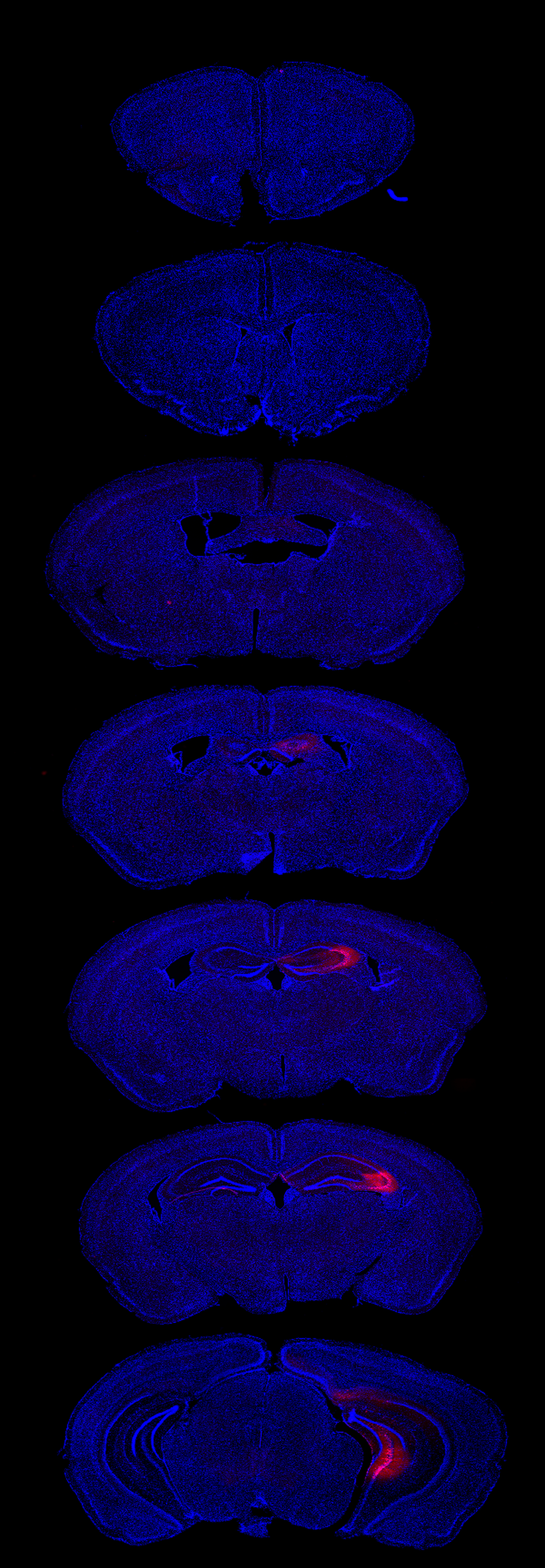

Supplement: Supplementary Figure 9 — AAV-PhP.eB-mediated transduction efficiently covers the dorsoventral extent of the hippocampal CA3 sub-region. Continuous coronal sections (30 μm, every 5th section) through the ipsilateral hippocampus, two weeks after stereotaxic injection of AAV-PhP.eB carrying an RFP reporter under the U6 promoter (titer ≈ 1 × 10¹³ vg/mL). Representative low-magnification confocal mosaic showing RFP signal (red) and DAPI (blue) across the rostrocaudal extent of CA3. Note continuous RFP coverage along the dorsoventral axis, confirming that the single stereotaxic injection produces anatomically broad transduction across the CA3 sub-region targeted by our behavioural and biochemical analyses (Figure 5 and 2). Scale bar, 200 μm. [file Image9.png]

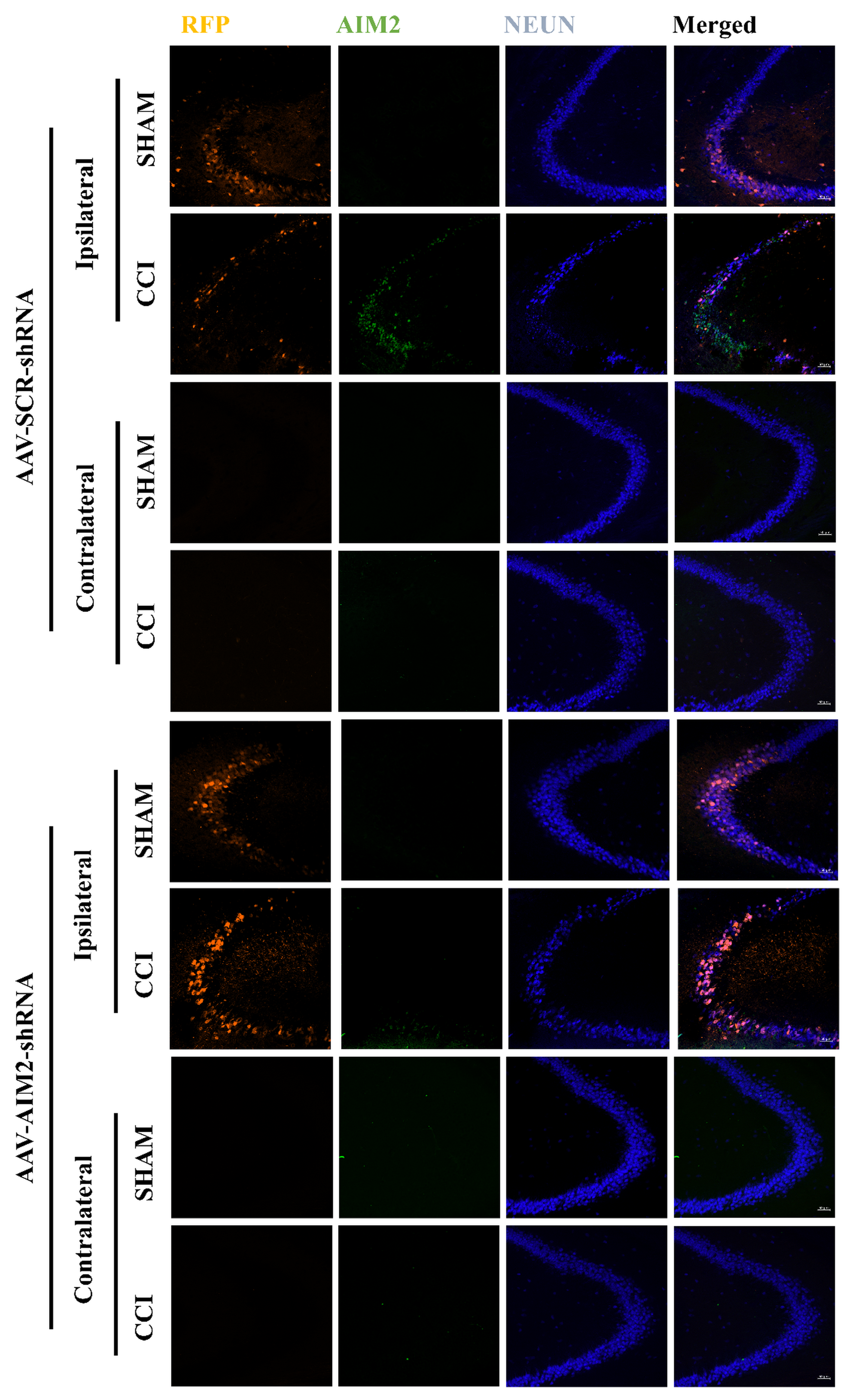

Supplement: Supplementary Figure 10 — Triple immunolabelling confirms neuron-restricted AIM2 knockdown and a preserved contralateral hemisphere after stereotaxic AAV-PhP.eB-shAIM2 injection. Mice received unilateral stereotaxic injection of AAV-PhP.eB-shAIM2 (RFP reporter; titer ≈ 1 × 10¹³ vg/mL) or AAV-PhP.eB-shScramble into the ipsilateral CA3 two weeks before CCI. Brains were collected 28 days post-CCI. (A) Representative confocal images of the ipsilateral CA3 region triple-labelled for the AAV reporter RFP (red), AIM2 (green) and the neuronal marker NeuN (blue). RFP co-localises predominantly with NeuN (>95% co-localisation in cell-type quantification), while in shAIM2-transduced (RFP-positive) neurons AIM2 immunoreactivity is markedly reduced or absent, demonstrating successful neuron-restricted knockdown. Scale bar, 50 μm (main panels); 20 μm (insets). (B) Representative confocal images of the contralateral hippocampal CA3 region from the same animals, showing no detectable AIM2 upregulation and preserved NeuN-positive neuronal density, supporting that the observed therapeutic effects are locally driven by the focal ipsilateral CCI and the unilateral AAV intervention. Scale bar, 50 μm. (C) Additional co-staining of RFP with the microglial marker Iba1 and the astrocytic marker GFAP (separate sections from the same brains) confirms negligible AAV transduction of microglia or astrocytes (<5% overlap), establishing the neuron-restricted tropism of the AAV-PhP.eB delivery used throughout this study. Scale bar, 50 μm. [file Image10.png]

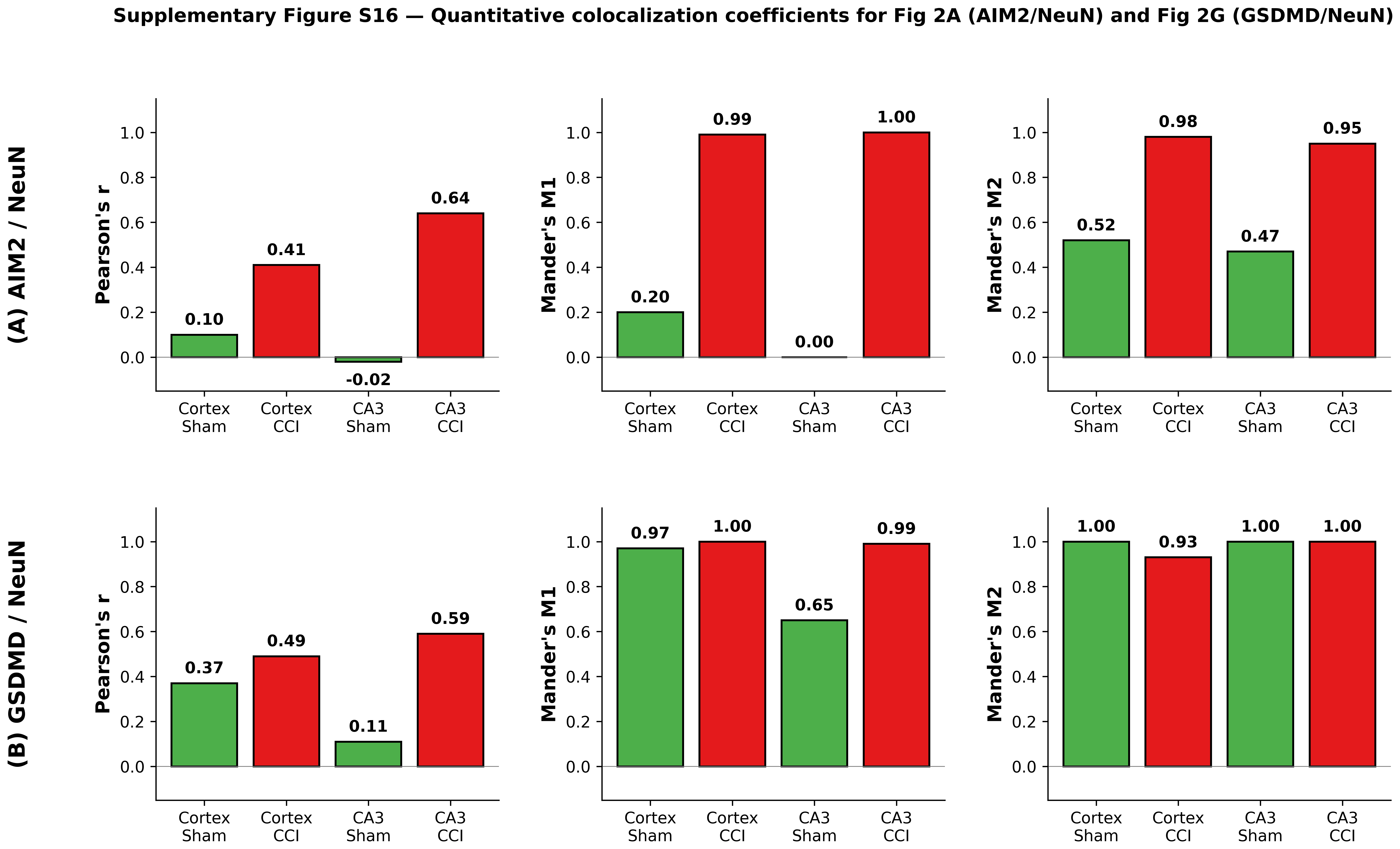

Supplement: Supplementary Figure 12 — Quantitative colocalization coefficients for Figure 3A (AIM2/NeuN) and Figure 3G (GSDMD/NeuN). Bar charts showing Pearson’s correlation coefficient (r) and Mander’s overlap coefficients (M1, M2), computed for the representative confocal images presented in main Figures 3A and G using a Python implementation of Costes’ regression-based automatic thresholding, validated against the Fiji Coloc2 plugin. (A) AIM2/NeuN coefficients across Cortex and CA3 (Sham vs CCI). (B) GSDMD/NeuN coefficients across Cortex and CA3 (Sham vs CCI). M1 = fraction of total AIM2/GSDMD signal located within NeuN-positive pixels (≈ 1.0 in CCI panels indicates near-complete neuronal localization). M2 = fraction of total NeuN signal that is co-positive for AIM2/GSDMD. Raw.czi files (n = 8), per-panel coefficient table, and the reproducible Python script are deposited as Figure 3 source data. [file Image12.png]
